# The Epigenetic Regulatory Protein CBX2 Promotes mTORC1 Signalling and Inhibits DREAM Complex Activity to Drive Breast Cancer Cell Growth

**DOI:** 10.3390/cancers14143491

**Published:** 2022-07-18

**Authors:** Lucie J. Bilton, Chloe Warren, Rebecca M. Humphries, Shannon Kalsi, Ella Waters, Thomas Francis, Wojtek Dobrowinski, Pedro Beltran-Alvarez, Mark A. Wade

**Affiliations:** 1 Faculty of Health Sciences, University of Hull, Hull HU6 7RX, UK; lucie.bilton@hull.ac.uk (L.J.B.); chloewarren1997@gmail.com (C.W.); r.humphries-2014@hull.ac.uk (R.M.H.); s.kalsi-2016@hull.ac.uk (S.K.); t.i.francis-2021@hull.ac.uk (T.F.); w.dobrowinski-2018@hull.ac.uk (W.D.); p.beltran-alvarez@hull.ac.uk (P.B.-A.); 2 Department of Life, Health and Chemical Sciences, The Open University, Milton Keynes MK7 6AA, UK; ella.waters@open.ac.uk

**Keywords:** epigenetics, breast cancer, TNBC, CBX2, polycomb, PRC1, mTORC1, RBL2

## Abstract

**Simple Summary:**

Cancer develops due to the expression of genes that promote cell growth and the repression of genes that limit growth. Epigenetics is a mechanism that regulates gene expression via the chemical modification of DNA and histones. Proteins that regulate this process have emerged as potential therapeutic targets. Here, we investigate the role of the epigenetic regulatory protein CBX2 in aggressive forms of breast cancer, which have few therapeutic options. We show that functioning CBX2 is crucial for cancer cell growth and viability. By analysing gene expression patterns in CBX2-depleted cells, we show that CBX2 activates signalling pathways that promote cell growth (mTORC1 signalling) and inhibits the activity of a protein complex that limits cell growth (the DREAM complex) by repressing the expression of key tumour suppressor genes. We have therefore identified novel mechanisms by which CBX2 promotes breast cancer growth and provide evidence that inhibition of CBX2 may be a novel therapeutic strategy.

**Abstract:**

Chromobox 2 (CBX2) is a chromatin-binding component of polycomb repressive complex 1, which causes gene silencing. CBX2 expression is elevated in triple-negative breast cancer (TNBC), for which there are few therapeutic options. Here, we aimed to investigate the functional role of CBX2 in TNBC. CBX2 knockdown in TNBC models reduced cell numbers, which was rescued by ectopic expression of wild-type CBX2 but not a chromatin binding-deficient mutant. Blocking CBX2 chromatin interactions using the inhibitor SW2_152F also reduced cell growth, suggesting CBX2 chromatin binding is crucial for TNBC progression. RNA sequencing and gene set enrichment analysis of CBX2-depleted cells identified downregulation of oncogenic signalling pathways, including mTORC1 and E2F signalling. Subsequent analysis identified that CBX2 represses the expression of mTORC1 inhibitors and the tumour suppressor RBL2. RBL2 repression, in turn, inhibits DREAM complex activity. The DREAM complex inhibits E2F signalling, causing cell senescence; therefore, inhibition of the DREAM complex via CBX2 may be a key oncogenic driver. We observed similar effects in oestrogen receptor-positive breast cancer, and analysis of patient datasets suggested CBX2 inhibits RBL2 activity in other cancer types. Therapeutic inhibition of CBX2 could therefore repress mTORC1 activation and promote DREAM complex-mediated senescence in TNBC and could have similar effects in other cancer types.

## 1. Introduction

Multiple subtypes of breast cancer (BCa) exist with tumours clinically stratified by the presence of oestrogen receptor (ER), progesterone receptor (PR) and HER2 proteins. For ER- and HER2-positive (+ve) subtypes, therapeutics that target the activity of these proteins have been very successful in reducing overall BCa deaths in the last 30 years [1,2]. For BCa that does not express ER, PR or HER2 (triple-negative breast cancer-TNBC), there is a paucity of therapeutic options. TNBC accounts for approximately 15% of all BCa cases, has a higher chance of recurrence within the first 3 years, is more aggressive, and has higher metastatic potential than other BCa subtypes [3]. The aggressive nature of TNBC, coupled with a lack of targeted therapeutics, means patients with this disease have a relatively poor prognosis (mortality rate within 5 years ~40%) [3]. There is, therefore, a crucial need for new and effective interventions to treat TNBC.

Polycomb group (PcG) proteins repress gene expression via two polycomb repressive complexes (Polycomb repressive complex 1 and 2; PRC1/PRC2) that modulate epigenetic post-translational histone modifications. PRC1 is comprised of different combinations of the polycomb (CBX2/4/6/7/8), polycomb group factor (PCGF1-6), human polyhomeotic homolog (HPH1-3) and E3-ligase (RING1/2) family of proteins. Canonical-PRC1 (cPRC1) contains one of each protein subunit; therefore, a diverse range of potential PRC1 complex compositions can be assembled. Classically, cPRC1 recognises histone H3 lysine (K) 27 tri-methylation (me3) (H3K27me3) via chromodomains within CBX proteins and catalyses mono-ubiquitination of H2AK119, resulting in chromatin compaction and silencing of target genes [4]. H3K27me3 is deposited by EZH2, which is a component of PRC2 [4]. Importantly, different cPRC1 complexes regulate distinct transcriptomic profiles, and the composition of the active form of cPRC1 is cell context-specific [4,5,6]. This suggests that different and specific cPRC1 complexes may be involved in cancer development and could therefore be targeted for therapy.

Expression of the cPRC1 protein chromobox 2 (CBX2) is elevated in a number of solid tumours, including colon, prostate, breast, stomach, glioblastoma (GBM), and lung cancer; indicating a potential oncogenic role for this protein [7,8,9,10,11,12]. In BCa, *CBX2* is elevated in aggressive tumours with a poor prognosis, and high *CBX2* expression is associated with reduced overall survival [7,13,14]. *CBX2* gene expression is highest in the basal-like breast cancer (BLBC) molecular subtype, of which 60–90% are TNBC [3,7,15]. Gene expression analysis of patient data sets has implicated CBX2 in metabolic reprogramming via regulation of mTORC1 signalling and PI3K/AKT pathway activation [15,16]; however, little mechanistic or functional study of CBX2 in TNBC has been reported [14,15,16].

Here we show that CBX2 is required for cell growth in multiple models of BCa. Through CBX2 knockdown rescue experiments and CBX2 chromodomain inhibitor treatment, we show that the chromatin-binding activity of CBX2 is crucial for TNBC growth and viability. RNA-sequencing analysis of CBX2-depleted cells has identified oncogenic processes promoted by CBX2 expression, and we show for the first time that CBX2 represses the expression of the tumour suppressor gene RBL2. We provide evidence that depletion of CBX2 increases RBL2 expression, which, in turn, enhances RBL2 recruitment to key pro-proliferative E2F-target genes to mediate gene silencing via dimerisation partner (DP), retinoblastoma (RB) link, E2F and MuvB (DREAM)-complex activity. Overall, this study provides mechanistic insights into the oncogenic role of CBX2 in TNBC and promotes CBX2 as a promising therapeutic target.

## 2. Materials and Methods

### 2.1. Cell Culture

MCF-7, T47D, MDA-MB-231, and Hs578T cells were provided by Dr. Luke Gaughan (Newcastle University) and the MDA-MB-468 cell line by Dr. Isabel Pires (University of Hull). All cell lines were authenticated by short tandem repeat analysis. MCF-7, T47D and MDA-MB-231 cells were cultured in RPMI-1640 media (Gibco) containing 10% foetal calf serum (FCS) (Gibco) and 1% penicillin/streptomycin (Merck). Hs578T and MDA-MB-468 cells were cultured in DMEM (Gibco) containing 10% foetal calf serum (FCS) (Gibco) and 1% penicillin/streptomycin (Merck).

### 2.2. Cell Line Transfection and CBX2 Inhibitor Treatments

For knockdown experiments, three CBX2-targeting siRNAs (siCBX2#1/2/3) and a non-silencing control siRNA (siSCR) were transfected into cells using Lipofectamine RNAiMAX (Invitrogen) to a final concentration of 25 nM, according to manufacturer recommendations. For overexpression experiments, cells were transfected 24 h after plating with 1 µg of plasmid using Lipofectamine 3000 (Invitrogen), according to manufacturer recommendations. For knockdown rescue experiments, cells were co-transfected 24 h after plating with 25 nM siRNA and 0.25 µg of plasmid using Lipofectamine 3000 (Invitrogen). For CBX2 inhibitor experiments, cells were treated at the time of plating with a DMSO vehicle control or SW2_152F (5 or 50 µM), ensuring that the final percentage of DMSO in culture media was consistent across all arms of the experiment.

### 2.3. Protein and Gene Expression Analysis

To assess the expression of CBX2, RBL2 and α-tubulin proteins, cells were harvested in SDS-sample buffer (10% β-mercaptoethanol, 125 mM Tris–HCl (pH 6.8), 2% SDS, 10% glycerol, 0.005% bromophenol blue) and assessed by Western blot as previously described [17] (antibody details Supplementary Table S2). Band intensity ratios for Western blot images were determined using ImageJ software and are detailed in Supplementary Information.

For gene expression analysis, RNA was extracted from transfected cells using the RNeasy Mini Kit (Qiagen) and cDNA was generated for analysis by quantitative-PCR (qPCR) using SYBR Green JumpStart TaqReady Mix (Merck) and a StepOne Plus Real-Time PCR machine (Applied Biosystems) (primer sequences in Supplementary Table S3). Gene expression relative to the siSCR control sample was calculated using the ΔΔ−Ct method using *RPL13A* gene expression as the normalisation control.

### 2.4. Cell Growth and Cell Cycle Analysis

Cells were grown for at least 72 h post transfection or SW2_152F treatment and monitored for cell growth by cell counts using a haemocytometer. The cell cycle phase was assessed by propidium iodide flow cytometry using a FACSCalibur (BD Biosciences) as previously described [18].

### 2.5. RNA Sequencing and Gene Set Enrichment Analysis

MDA-MB-231 and MCF-7 cells were transfected with either siSCR (both cell lines), siCBX2#2 (MDA-MB-231 only) and siCBX2#3 (both cell lines) as described above and grown for 72 h prior to RNA extraction using the RNeasy Mini Kit (Qiagen). Library preparation and sequencing using the Illumina NovaSeq 6000 platform were performed by Novogene Co. Ltd. (Cambridge, UK). Read alignment was performed using the HISAT2 algorithm and FPKM calculated to estimate gene expression in each sample [19]. Gene expression data from the siSCR transfected samples were used as the reference gene expression data set. Differential gene expression analysis was performed using read counts obtained from gene expression analysis and the DESeq2 R package [20]. Gene expression change in cells transfected with siCBX2#2/3 compared to cells transfected with siSCR was log_2_ transformed, and the *p*-values calculated were adjusted using the Benjamini and Hochberg approach for controlling the false discovery rate (padj). Only genes significantly (padj < 0.05) up- or downregulated 1.5-fold (log_2_ fold change = ±0.585) with a gene symbol/ID annotation were used in subsequent analysis (classified as “CBX2-regulated genes”). All RNA sequencing data discussed in this publication have been deposited in NCBI’s Gene Expression Omnibus (GEO-https://www.ncbi.nlm.nih.gov/geo/ accessed 11 March 2022) and are accessible through the GEO Series accession number (GSE198420).

CBX2 regulated genes were analysed using gene set enrichment analysis (GSEA) software (Broad Institute, Cambridge, MA, USA; [21,22]). Genes were ranked based on their log_2_ fold change (most positive to most negative). Ranked lists were then compared against the “Hallmark”, “GO”, and “Oncogenic” curated gene sets from the Molecular Signatures Database v6.2 (MSigDB, Broad Institute). 

### 2.6. Chromatin Immunoprecipitation-qPCR (ChIP-qPCR) and Cleavage under Targets and Release Using Nuclease (CUTandRUN)-qPCR

For ChIP-qPCR experiments, the ChIP procedure as described in [23] was followed. Briefly, MDA-MB-231 and MDA-MB-468 cells were transfected with 25 nM siSCR or 25 nM of a siCBX2 pool consisting of equal amounts of siCBX2#1/2/3 and grown for 48 h. Chromatin was harvested and sonicated using a Bioruptor (Diagenode). ChIP was performed on 10 µg of chromatin using 10 µL of anti-RBL2 antibody (Cat#13610; Cell Signaling Technologies). The ChIP protocol comprised of a 6 h antibody coating of Dynabeads (Invitrogen) and a 10 h immunoprecipitation incubation. DNA from ChIP samples and input samples (chromatin from the original sonicated sample) were purified, and qPCR was performed using primers specific to *CCNA2*, *CCNB1* and *UBE2C* gene promotors (primer sequences in Supplementary Table S3). Data were presented as mean fold difference in percentage input relative to the percentage input calculated for siSCR transfected cells.

For cleavage under targets and release using nuclease (CUT&RUN) qPCR experiments, the CUT&RUN assay kit (Cell Signaling Technologies) was used following the manufacturer’s instructions. Briefly, 100,000 MDA-MB-231 cells were used per CUT&RUN reaction with 2 µg of anti-CBX2 (Cat#C15410339; Diagenode) or isotype IgG control (Diagenode) antibodies. For each experiment, input samples were generated from the same cell population by extracting and sonicating DNA from 100,000 cells. Purified CUT&RUN-DNA and input samples were subject to qPCR using primers specific to *TSC1*, *PRKAA2* and *RBL2* gene promotors (primer sequences in Supplementary Table S3). Data were calculated as percentage input and presented as the mean fold difference of percentage input relative to percentage input in the IgG control arm of the experiment.

### 2.7. CBX2 Correlation Analysis of Patient Data Sets

Breast Invasive Carcinoma, Lung Adenocarcinoma and GBM TCGA PanCancer Atlas data sets were analysed using the cBio Cancer Genomics Portal [24,25] to identify genes positively and inversely correlated with CBX2 expression in patient tumours. Genes were ranked via their Spearman’s correlation value (most positive to most negative), and genes with a Spearman’s correlation *p*-value > 0.05 were excluded. The subsequent CBX2 correlated gene lists were analysed using GSEA as described above.

## 3. Results

### 3.1. CBX2 Chromatin Interactions Are Required for TNBC Cell Growth and Viability

**Figure 1:**
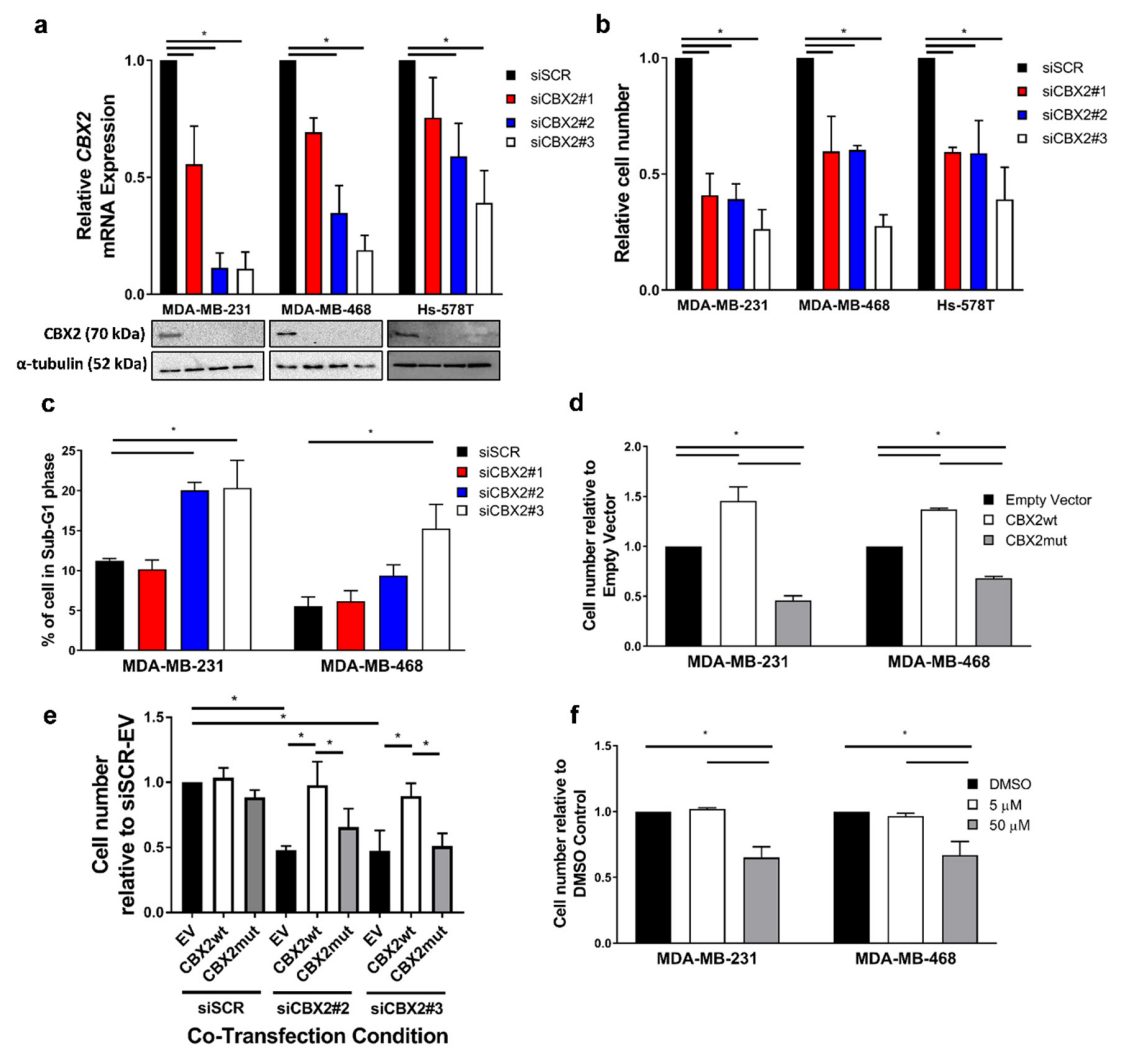
CBX2 chromatin interactions are required for TNBC cell growth/viability. MDA-MB-231, MDA-MB-468 and Hs578T cells were transfected with either a non-silencing siRNA control (siSCR) or one of 3 *CBX2* targeting siRNAs (siCBX2#1/#2/#3). Cells were cultured for 72 h, followed by: gene and protein expression analysis (**a**), cell growth analysis (**b**) and cell cycle analysis (**c**). (**a**) CBX2 gene and protein expression was assessed by qPCR and Western blot, respectively. α-tubulin protein expression was assessed to compare protein loading between samples. Lanes on the Western blot images align with the cell line and experimental arm indicated in the graph above. *CBX2* gene expression data are the mean of 3 repeats ± SEM and are expressed relative to *CBX2* expression in siSCR transfected cells. (**b**) Cell growth was assessed by cell counts. Cell count data are the mean of 3 repeats ± SEM and are expressed relative to cell counts for siSCR transfected cells. (**c**) The effect of CBX2 knockdown on the cell cycle was assessed by propidium iodide flow cytometry. The % of cells in the sub-G1 phase of the cell cycle is shown and is an average of 3 repeats ± SEM. (**d**) MDA-MB-231 and MDA-MB-468 cells were transfected with 1 µg of either an empty vector control plasmid or a plasmid containing a wild-type version of CBX2 (CBX2wt), or a chromodomain-deficient mutant (CBX2mut), and grown for 72 h followed by cell counts. Cell count data are the mean of 3 repeats ± SEM and are expressed relative to cell counts for empty vector-transfected cells. (**e**) MDA-MB-231 cells were co-transfected with combinations of siSCR, siCBX2#2/3 and empty vector (EV) or CBX2wt/mut plasmids and grown for 72 h followed by cell counts. Cell count data are the mean of 3 repeats ± SEM and are expressed relative to cell counts for siSCR and empty vector co-transfected cells. (**f**) MDA-MB-231 and MDA-MB-468 cells were treated with a DMSO control or 5 and 50 µM of SW2_ 152F and grown for 72 h (MDA-MB-231) or 96 h (MDA-MB-468) followed by cell counts. Cell count data are the mean of 3 repeats ± SEM and are expressed relative to cell counts in the DMSO control arms of the experiment. In all experiments *p*-values were determined by Turkey’s multiple comparisons test (* denotes *p* < 0.05).

A pro-proliferative role for CBX2 in TNBC has only been shown in a single cell line (MDA-MB-231) [15]. Here, we depleted CBX2 in three TNBC cell lines (MDA-MB-231, Hs578T and MDA-MB-468) using three independent siRNA sequences (siCBX2#1/2/3) to determine if CBX2 is required for TNBC cell growth across multiple models. Knockdown of CBX2 gene and protein expression was confirmed in each cell line by qPCR and Western blot, respectively (Figure 1a). In each case, knockdown of *CBX2* mRNA was most efficient using siCBX2#2 and #3 compared to siCBX2#1; however, protein expression of CBX2 was reduced below a detectable level following transfection with all three siRNAs.

Knockdown of CBX2 reduced the number of cells in each cell line 72 h post transfection compared to a non-silencing siRNA control (siSCR) (Figure 1b), indicating a requirement for cell growth. Cell cycle analysis of MDA-MB-231 and MDA-MB-468 cells showed that knockdown of CBX2 caused an increase in cells in the sub-G1 phase, indicating an increase in cell death (Figure 1c). This was observed particularly for cells transfected with siCBX2#2 and #3 compared to siCBX2#1, which may reflect the comparative efficiency of *CBX2* mRNA knockdown using these siRNAs (Figure 1a). Knockdown of CBX2 also caused an accumulation of cells in the G2/M phase of the cell cycle (Supplementary Figure S1).

To investigate the effect of CBX2 overexpression on cell growth, MDA-MB-231 and MDA-MB-468 cells were transfected with previously validated plasmids containing either a wild-type CBX2 construct (pCMV-CBX2wt) or a chromodomain-deficient mutant (pCMV-CBX2mut) [26]. Wild-type CBX2 overexpression increased cell numbers 72 h post transfection compared to cells transfected with empty vector control (Figure 1d; Supplementary Figure S2). Conversely, overexpression of the chromodomain-deficient mutant depleted cell numbers (Figure 1d), which indicated a dominant negative effect and suggested that the chromatin-binding activity of CBX2 was crucial for cell growth. To validate this, MDA-MB-231 cells were co-transfected with either siSCR, siCBX2#2 or siCBX2#3 and either the empty vector, pCMV-CBX2wt or pCMV-CBX2mut. Cell counts 72 h post transfection showed that wild-type CBX2 elevated cell numbers in siCBX2#2/3 transfected cells to the levels observed in siSCR transfected cells (Figure 1e). The chromodomain-deficient mutant was unable to elevate cell numbers following CBX2 depletion. This rescue effect was also shown in MDA-MB-468 cells co-transfected with either siSCR or siCBX2#3 and relevant plasmids (Supplementary Figure S3), indicating that CBX2 chromatin interactions were crucial for TNBC cell growth.

As further validation, MDA-MB-231 and MDA-MB-468 cells were treated with the CBX2 inhibitor SW2_152F, which blocks CBX2 chromatin interactions [27]. SW2_152F treatment did not significantly affect protein expression of CBX2 (Supplementary Figure S4); however, MDA-MB-231 and MDA-MB-468 cell numbers were reduced 72 and 96 h, respectively, post treatment with 50 µM of inhibitor compared to vehicle control (Figure 1f). Together with knockdown rescue experiments, the data indicate that CBX2 chromatin interactions are required for TNBC cell growth/viability and provides, for the first time, proof of concept that pharmacological inhibition of CBX2-chromatin interactions can inhibit TNBC cell growth.

### 3.2. CBX2 Promotes Proliferative and Oncogenic Gene Signatures

CBX2 has been implicated in regulating PI3K/AKT and mTORC1 oncogenic signalling pathways in BCa; however, these pathways were identified following correlative analysis of *CBX2* gene expression in patient samples rather than by direct transcriptomic analysis [15,16]. To identify genes and biological processes regulated by CBX2 in TNBC, MDA-MB-231 cells were transfected with either siSCR or siCBX2#3 and grown for 72 h prior to RNA sequencing. The CBX2 regulated transcriptome was defined as genes significantly differentially expressed 1.5-fold following CBX2 knockdown and consisted of 2884 upregulated and 1060 downregulated genes (padj < 0.05).

**Figure 2:**
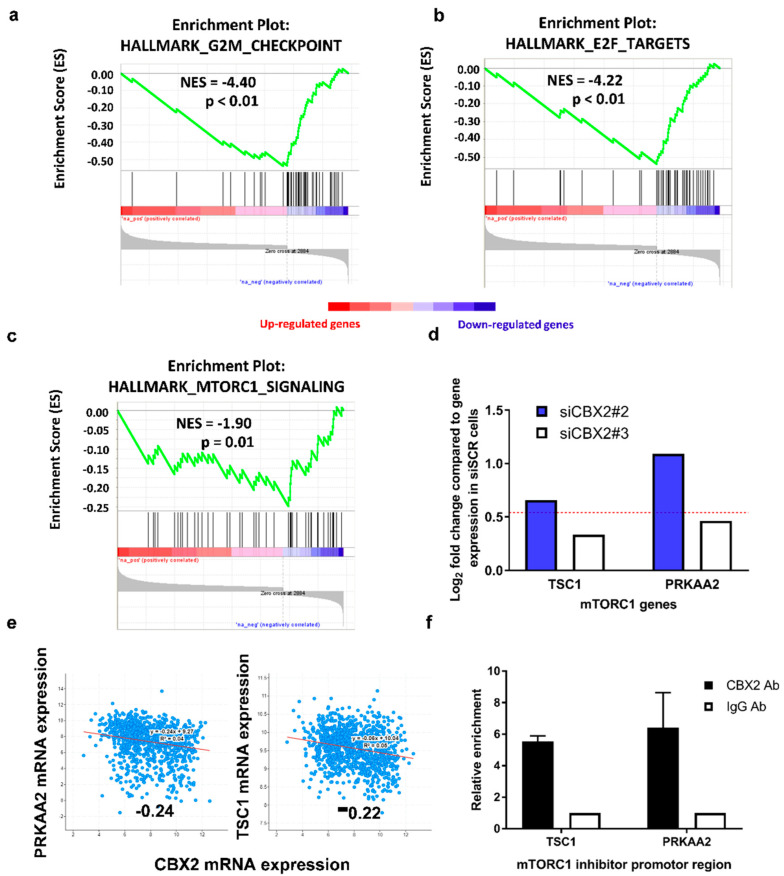
The CBX2 regulated gene signature is associated with pro-proliferative and oncogenic signalling pathways. (**a**–**d**) MDA-MB-231 cells were transfected with either a non-silencing control siRNA (siSCR) or siCBX2#3 and grown for 72 h (*n* = 3). RNA was extracted, and RNA sequencing analysis was performed. GSEA of the CBX2-regulated transcriptome (defined as genes significantly (padj < 0.05) up (*n* = 2884) or down (*n* = 1060) regulated 1.5-fold after knockdown compared to the siSCR control) against the “Hallmark” curated data set identified significant negative enrichment of genes associated with HALLMARK_G2M_CHECKPOINT (**a**), HALLMARK_E2F_TARGETS (**b**) and HALLMARK_MTORC1_SIGNALING (**c**) gene sets. NES = Normalised Enrichment Score. p = nominal *p*-value. (**d**) RNA-seq gene expression analysis of *TSC1* and *PRKAA2* in cells transfected with siCBX2#2 and #3 compared to siSCR transfected cells. Data are presented as log_2_ fold change in expression. The red line represents a 1.5-fold increase (log_2_ fold change = 0.585) in expression compared to siSCR transfected cells. Both genes are significantly differentially expressed in CBX2 depleted cells compared to siSCR transfected cells (padj < 0.05). (**e**) Correlation of *CBX2* and *PRKAA2*/*TSC1* gene expression in the TCGA, PanCancer Atlas Breast Invasive Carcinoma data set. Values = Spearman co-efficient. *p* < 0.05. (**f**) CUT&RUN qPCR analysis of promoter regions of *TSC1* and *PRKAA2* in MDA-MB-231 cells using antibodies specific to CBX2 and isotype control (IgG). Data are an average of 2 independent experiments ± SEM and are expressed relative to the level of enrichment measured in the IgG control.

Gene Set Enrichment Analysis (GSEA) [21] of the CBX2 regulated transcriptome identified that downregulated genes were enriched for genes also downregulated when other components of the PRC1/PRC2 regulatory axis are abrogated (such as EZH2 and BMI1; Table 1). This validated that knockdown of CBX2 affected PcG-mediated gene regulation in MDA-MB-231 cells. GSEA also identified that downregulated genes were involved in cell proliferation and progression through the G2/M cell cycle checkpoint (Figure 2a; Table 1), which correlates with cell growth and cell cycle effects seen following CBX2 depletion (Figure 1b; Supplementary Figure S1).

Downregulated genes were also enriched in gene sets associated with oncogenic pathways, including E2F target genes (NES = −4.22; *p* < 0.01), genes that are upregulated through activation of the mTORC1 complex (NES = −1.9; *p* = 0.01), and MYC target genes (NES = −1.85; *p* = 0.01) (Figure 2b,c; Supplementary Figure S5), indicating that CBX2 is required to promote these signalling pathways. To validate these findings, RNA-seq and GSEA was also performed on cells transfected with siCBX2#2, which identified that downregulated genes were significantly enriched in similar processes (Supplementary Table S4), including G2/M checkpoint (NES = −2.62; *p* < 0.01), E2F targets (NES = −1.77; *p* = 0.01), MYC targets (NES = −2.22; *p* < 0.01) and mTORC1 signalling (NES = −2.43; *p* < 0.01).

mTORC1 activation promotes tumour formation, proliferation and metastasis and is predominantly associated with cell growth and metabolism [28]. Downregulation of an active mTORC1 gene signature in CBX2 depleted MDA-MB-231 cells supports previous analysis of patient datasets that suggested CBX2 promotes mTORC1-signalling [28]. This previous study also showed that CBX2 knockdown in MDA-MB-231 decreased phosphorylation of ribosomal S6 protein, a downstream target of mTORC1. The precise role of CBX2 in mTORC1 signalling, however, has not been determined. Considering the classic transcriptional repressive role of cPRC1, we analysed the RNA-seq data set for upregulation of genes that code for known inhibitors of mTORC1 activation. *PRKAA2* and *TSC1*, which code for proteins that repress mTORC1 [29], were significantly upregulated following CBX2 knockdown (Figure 2d). *CBX2* expression is also inversely correlated with *PRKAA2* and *TSC1* expression in BCa patient data sets (Figure 2e). This led to the hypothesis that CBX2 may repress *PRKAA2* and *TSC1* expression in TNBC to promote mTORC1 signalling. CUT&RUN qPCR using an anti-CBX2 antibody in MDA-MB-231 cells showed enrichment of CBX2 at the promoter regions of these genes compared to an IgG control, suggesting that CBX2 may directly cause repression of *PRKAA2* and *TSC1* via cPRC1-mediated activity (Figure 2f). The effect of CBX2 knockdown on PRKAA2 and TSC1 protein expression still needs to be evaluated; however, this analysis has potentially identified a previously unknown mechanism for CBX2-mediated activation of mTORC1 signalling.

### 3.3. CBX2 Inhibits RBL2 Expression and DREAM Complex Activity to Promote TNBC Cell Growth

E2F activity promotes cell growth and progression through the cell cycle, and expression of E2F target genes is elevated in many cancers [30]. E2F target genes were significantly downregulated following CBX2 depletion, as were genes repressed by the tumour suppressor RBL2 (p130) (Supplementary Figure S6). RBL2 (or its paralogue RBL1 (p107)) are part of the dimerisation partner (DP), retinoblastoma (RB)-link, E2F and MuvB (DREAM)-complex, which binds to E2F binding sites to repress E2F target gene expression and cause cell cycle arrest [31]. Activation of RBL2-DREAM complex activity following CBX2 depletion was therefore hypothesised to inhibit E2F target gene expression and TNBC cell growth.

**Figure 3:**
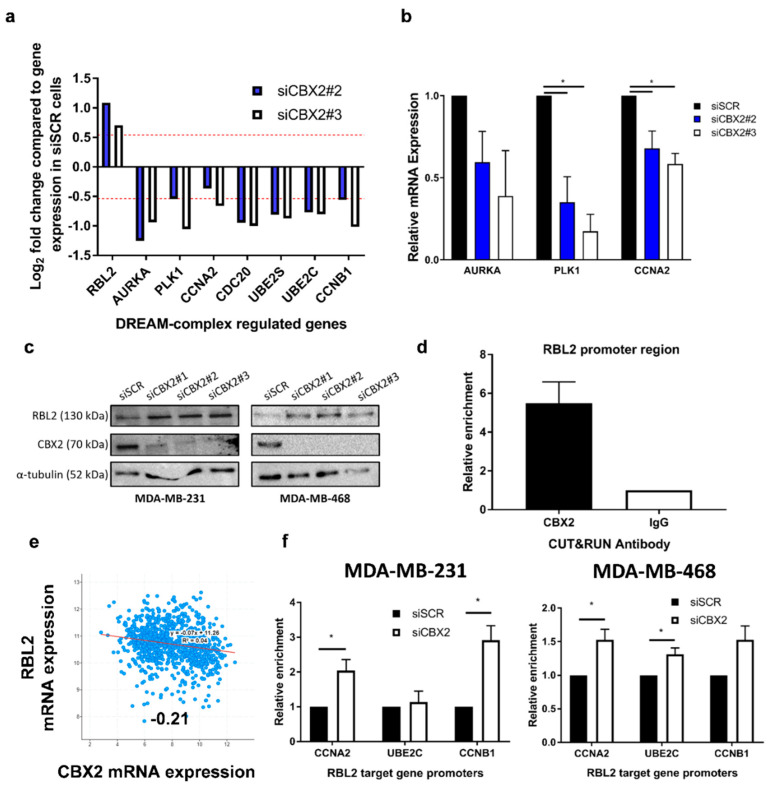
CBX2 inhibits expression of the tumour suppressor gene *RBL2* to promote cell growth in TNBC. (**a**) RNA-seq analysis of RBL2-DREAM complex target genes in MDA-MB-231 cells transfected with siCBX2#2 and #3 compared to siSCR transfected cells (*n* = 3). Data are presented as log_2_ fold change in expression. The red line represents a 1.5-fold increase or decrease in expression compared to siSCR transfected cells. All genes are significantly differentially expressed in CBX2 depleted cells compared to siSCR transfected cells (padj < 0.05). (**b**) MDA-MB-468 cells were transfected with either a non-silencing control siRNA (siSCR) or siCBX2#2/#3 and grown for 72 h, followed by RNA extraction and qPCR analysis of RBL2-DREAM complex target genes. qPCR data are an average of 3 repeats ± SEM and are expressed relative to gene expression in siSCR transfected cells. *p*-values were determined by Student’s *t*-test comparing CBX2 depleted cells to siSCR transfected cells (* denotes *p* < 0.05). (**c**) MDA-MB-231 and MDA-MB-468 cells were transfected with siRNA and grown for 72 h, followed by protein extraction and Western blot analysis using antibodies specific to RBL2, CBX2 and α-tubulin. α-tubulin was used to compare protein loading between samples. (**d**) CUT&RUN qPCR analysis of the promoter region of *RBL2* in MDA-MB-231 cells using antibodies specific to CBX2 and isotype control (IgG). Data are an average of 2 independent experiments ± SEM and are expressed relative to the level of enrichment measured in the IgG control. (**e**) Correlation of *CBX2* and *RBL2* gene expression in the TCGA, PanCancer Atlas Breast Invasive Carcinoma data set. Value = Spearman co-efficient. *p* < 0.05. (**f**) ChIP-qPCR analysis of RBL2-DREAM complex target gene promotors in MDA-MB-231 and MDA-MB-468 cells transfected with either siSCR or a siCBX2 targeting pool using an antibody specific to RBL2. Data are an average of 3 independent experiments ± SEM and are expressed relative to the level of enrichment measured in the siSCR transfected cells. *p*-values were determined by Student’s *t*-test comparing CBX2 depleted cells to siSCR transfected cells (* denotes *p* < 0.05).

RNA-seq analysis showed that expression of *RBL2* was elevated in CBX2-depleted MDA-MB-231 cells alongside downregulation of known RBL2-DREAM complex target genes (Figure 3a) [32]. qPCR analysis of CBX2-depleted MDA-MB-468 cells also showed a decrease in RBL2-DREAM complex target gene expression, indicating that CBX2 knockdown may activate the DREAM complex in different TNBC models (Figure 3b). Western blot analysis showed upregulation of RBL2 protein expression in both cell lines following CBX2 knockdown (Figure 3c). CUT&RUN qPCR analysis showed that CBX2 was enriched at the *RBL2* promoter in MDA-MB-231 cells compared to IgG control (Figure 3d). Together, these data indicated that CBX2 may directly repress RBL2 expression. In support of this, *CBX2* expression is inversely correlated with *RBL2* expression in BCa patient samples (Figure 3e), and GSEA of the TCGA PanCancer BCa patient data set showed that *CBX2* expression positively correlates with E2F target gene expression (Supplementary Figure S7).

To investigate if knockdown of CBX2 and subsequent upregulation of RBL2 may directly cause repression of RBL2-DREAM complex target genes and contribute to observed phenotypic and gene expression changes in TNBC, ChIP-qPCR using an anti-RBL2 antibody was performed at promotors of RBL2 target genes involved in G2/M cell cycle progression that were downregulated following CBX2 depletion (*CCNA2*, *CCNB1* and *UBE2C*; Figure 3f). CBX2 depletion increased RBL2 enrichment at *CCNA2* (2.0-fold; *p* = 0.03), and *CCNB1* (2.9-fold; *p* = 0.01) promotors in MDA-MB-231 cells and *CCNA2* (1.5-fold; *p* = 0.03), *UBE2C* (1.3-fold; *p* = 0.03) and *CCNB1* (1.5-fold; *p* = 0.06) promotors in MDA-MB-468 cells (Figure 3f). This indicates that upregulation of RBL2 caused by CBX2 knockdown may increase DREAM complex recruitment to target gene promoters, which could subsequently inhibit cell cycle progression. This analysis has therefore identified a mechanism by which the tumour suppressor RBL2 is repressed in TNBC and proposes that CBX2 promotes TNBC cell growth via inhibition of DREAM complex activity.

### 3.4. The CBX2-RBL2 Regulatory Axis May Be Common across Multiple Cancer Types

**Figure 4:**
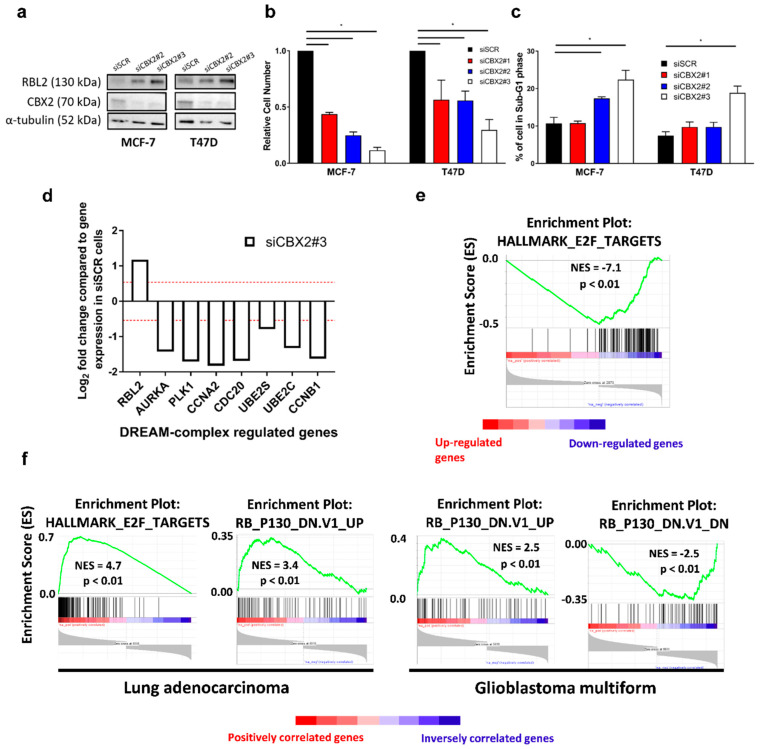
CBX2 is associated with DREAM complex activity in other cancer types. MCF-7 and T47D cells were transfected with either a non-silencing siRNA control (siSCR) or *CBX2* targeting siRNAs (siCBX2#1/#2/#3) and grown for 72 h followed by protein expression analysis (**a**), cell growth analysis (**b**) and cell cycle analysis (**c**). (**a**) CBX2 and RBL2 protein expression was assessed by Western blot. α-tubulin protein expression was assessed to compare protein loading between samples. (**b**) Cell growth was assessed by cell counts. Cell count data are the mean of 3 repeats ± SEM and are expressed relative to cell counts for siSCR transfected cells. (**c**) The effect on the cell cycle was assessed by propidium iodide flow cytometry. Data show the % of cells in the sub-G1 phase of the cell cycle and are an average of 3 repeats ± SEM. *p*-values for (**b**,**c**) were determined by Turkey’s multiple comparisons test (* denotes *p* < 0.05). (**d**) RNA-seq analysis of RBL2-DREAM complex target genes in MCF-7 cells transfected with siCBX2#3 compared to siSCR transfected cells. Data are presented as log_2_ fold change in expression. The red line represents a 1.5-fold increase or decrease in expression compared to siSCR transfected cells. All genes are significantly differentially expressed (padj < 0.05). (**e**) GSEA of the CBX2-regulated transcriptome (defined as genes significantly (padj < 0.05) up (*n* = 2973) or down (*n* = 2011) regulated 1.5-fold after knockdown compared to siSCR controls) against the “Hallmark” curated data sets identified significant negative enrichment of genes associated with HALLMARK_E2F_TARGETS. NES = normalised enrichment score. p = nominal *p*-value. (**f**) GSEA of genes significantly positively and inversely correlated (Spearman’s *p* < 0.05) with CBX2 expression from TCGA PanCancer Database for Lung Adenocarcinoma and GBM. NES = normalised enrichment score. p = nominal *p*-value.

To determine if CBX2 represses RBL2 expression in other BCa subtypes, we assessed the effect of CBX2 knockdown in ER+ve BCa cell lines (MCF-7 and T47D). As for TNBC, CBX2 knockdown increased RBL2 protein expression (Figure 4a), reduced cell numbers (Figure 4b) and caused an increase in sub-G1 cells, indicative of cell death (Figure 4c). Knockdown also reduced the number of cells in S-phase in both MCF-7 and T47D cell lines showing a growth inhibitory effect (Supplementary Figure S8). RNA-seq analysis of CBX2-depleted MCF-7 cells confirmed that *RBL2* was significantly upregulated and that RBL2-DREAM complex regulated genes were downregulated following CBX2 knockdown (Figure 4d). GSEA analysis of the MCF-7 CBX2-regulated transcriptome (2973 upregulated genes and 2011 downregulated genes) also showed that the E2F target gene set (NES = −7.1; *p* < 0.01) was downregulated following CBX2 depletion, as in TNBC, (Figure 4e; Supplementary Table S5), suggesting that the observed phenotypic effects may also be mediated by enhanced DREAM complex activity.

It is also worth noting that downregulation of the mTORC1-activated gene signature was observed following CBX2 knockdown in MCF-7 cells, as was a significant increase in expression of the mTORC1 inhibitor *TSC1* (Supplementary Table S5; Supplementary Figure S9). Both of these effects were also seen in TNBC, as has been discussed previously (Figure 2).

In addition to BCa, CBX2 expression is elevated in GBM and lung adenocarcinoma compared to normal tissue [10,33]. GSEA of genes that correlate with high *CBX2* expression in the TCGA PanCancer database for these cancer types shows positive enrichment for E2F target genes and for genes upregulated when expression of *RBL2* is low (Figure 4f). In turn, genes downregulated when expression of *RBL2* is low inversely correlates with high *CBX2* expression (Figure 4f). While further investigation is required, these data do suggest that CBX2 may inhibit the expression of the tumour suppressor RBL2 in multiple cancer types and, therefore, may repress RBL2-DREAM complex activity across multiple malignancies to promote cell growth.

## 4. Discussion

CBX2 is upregulated and implicated in the growth of a number of cancer types, suggesting it may be a potential therapeutic target [7,8,9,10,11,12,15,33,34,35]. In BCa, CBX2 is particularly upregulated in TNBC, which is an aggressive BCa subtype with a poor prognosis and few therapeutic options. Previous work has shown a growth inhibitory effect of CBX2 knockdown in single cell lines from both ER+ve (MCF-7 cells) and TNBC (MDA-MB-231 cells) BCa subtypes [14,15,16]; however, the mechanistic and functional role of CBX2 in TNBC has not yet been extensively studied. Our study has shown across multiple TNBC models that knockdown of CBX2 reduces cell number, can cause arrest in the G2/M phase of the cell cycle, and causes cell death, providing further evidence that CBX2 is required for TNBC cell growth and viability. Importantly, the dominant negative effect observed after overexpression of a chromodomain-deficient CBX2 mutant, coupled with experiments showing that reduction in cell numbers in CBX2-depleted cells could be rescued by the ectopic expression of the wild type, but not mutant, form of CBX2, has determined the importance of CBX2 chromatin binding for TNBC cell growth for the first time. Demonstrating a direct relationship between CBX2-chromatin interactions and TNBC cell growth or viability is a crucial target validation step for the potential therapeutic use of inhibitors that block this interaction.

Evidence suggests that CBX2 may play a more prominent role in cancer progression compared to other CBX paralogues. For example, CBX2 and CBX7 have been shown to have contrasting roles in BCa metabolism and cell growth, with CBX2 being associated with aggressive tumour subtypes and poorer patient outcomes [16]. Targeting CBX2 specifically may therefore be of clinical benefit. We have shown for the first time that treatment of TNBC cells with a selective CBX2 inhibitor, SW2_152F, reduces cell numbers. SW2_152F is cell permeable, targets the CBX2 chromodomain, and shows 24- to 1000-fold selective inhibition over other CBX proteins [27]. SW2_152F has been shown to inhibit the growth of neuroendocrine prostate cancer cells and to abrogate CBX2 chromatin interactions. The development of this inhibitor provides proof of principle that CBX2 can be targeted specifically. This study shows for the first time that pharmacological inhibition of CBX2-chromatin interactions can inhibit TNBC cell growth. Analysis of the effect of CBX2 inhibitors in in vivo and ex vivo BCa models would provide further validation of selective CBX2 inhibition as a therapeutic approach.

CBX2, as part of cPRC1, is involved in epigenetic regulation of gene expression, classically causing chromatin compaction and silencing of target genes [4]. CBX2 is implicated in regulating PI3K/AKT and mTORC1 oncogenic signalling pathways in BCa; however, these pathways were identified following correlative analysis of CBX2 gene expression in patient samples rather than by direct transcriptomic analysis [15,16]. In this study, we identified a putative CBX2-regulated transcriptome by RNA-seq analysis of CBX2-depleted cells. Downregulation of genes involved in cell proliferation and cell cycle progression correlated with changes to cell phenotype observed following CBX2 knockdown. Downregulation of genes expressed when mTORC1-signalling is active supported previous observations that CBX2 regulates mTORC1 activity [16]. For the first time, we identified that CBX2 knockdown upregulates expression of the mTORC1 inhibitors *TSC1* and *PRKAA2* and that CBX2 is bound to *TSC1* and *PRKAA2* promoters in MDA-MB-231 cells. TSC1 and TSC2 form a GTPase-activating protein complex that inhibits RHEB, a crucial activator of mTORC1 signalling [29]. Inactivating TSC1 mutations are associated with tumour formation due to mTORC1 overactivity [36]. Silencing of *TSC1* expression by CBX2 could therefore have similar oncogenic properties. *PRKAA2* codes for the catalytic subunit of the AMP-activated protein kinase (AMPK), which also inhibits mTORC1 activity via phosphorylation of both TSC2 (activating its GTPase activity) and RAPTOR, a key component of the active mTORC1 complex, phosphorylation of which prevents its association with mTORC1 [37,38]. Downregulation of AMPK activity has been reported to be a driver of cancer growth, and re-activation of AMPK signalling has been touted as a therapeutic option for BCa [39]. We, therefore, propose a novel mechanism by which CBX2 promotes mTORC1 activity via repression of mTORC1 inhibitors and the possibility that inhibition of CBX2 could reduce BCa cell growth and viability through downstream inhibition of mTORC1.

GSEA did not identify PI3K/AKT-signalling as a pathway significantly affected by CBX2 depletion in our study. This was surprising given the fact that PI3K/AKT-signalling has also been associated with CBX2 in advanced prostate cancer and GBM [8,33]. We did not look specifically at biomarkers of PI3K/AKT-signalling in our study, and we cannot rule out that this pathway is affected by CBX2 depletion; however, we found no transcriptomic evidence that this pathway was altered. PI3K/AKT is a positive regulator of mTORC1 signalling [40]; therefore, if CBX2 does promote the PI3K/AKT pathway in BCa, this may be another mechanism by which CBX2 regulates mTORC1 activity.

RNA sequencing analysis also identified that E2F target gene expression was downregulated following CBX2 depletion. E2F signalling is required for transition between all phases of the cell cycle and, therefore, cell growth [30]. In addition, an RBL2-regulated gene signature was activated upon CBX2 depletion in MDA-MB-231 cells, and RBL2 gene and protein expression increased following CBX2 knockdown. RBL2 is a putative tumour suppressor gene that constitutes part of the DREAM complex. The DREAM complex represses E2F target gene expression and inhibits cell cycle progression, ultimately causing cell senescence [31]. RBL2 has been shown to suppress tumour growth in vivo [41], and downregulation of *RBL2* has been reported in a number of cancers [42,43,44,45,46]. In BCa, epigenetic silencing at the *RBL2* transcriptional start site has been reported, although the proteins mediating this repression have not been identified [45,46]. We found an enrichment of CBX2 at the *RBL2* gene promoter, which suggests that CBX2-associated cPRC1 could directly repress *RBL2* expression. This study has therefore potentially identified a novel mechanism of *RBL2* silencing in BCa. Knockdown of CBX2 also increased RBL2 enrichment at a subset of DREAM complex target genes, indicating that repression of RBL2 expression by CBX2 inhibits DREAM complex activity to promote cell cycle progression and cell proliferation. Inhibition of CBX2 may therefore prevent tumour growth via re-activation of DREAM complex-mediated cell senescence.

Despite the relatively suitable therapeutic provision for hormone receptor-positive breast cancers, resistance to targeted therapy is common, after which tumours are difficult to treat [1,2]. We also assessed the effect of CBX2 knockdown in ER+ve BCa cell models and identified that CBX2 depletion reduced cell numbers and increased cell death, as has been seen previously [15,16]. Interestingly, through global transcriptomic investigation of MCF-7 cells, we also identified that mTORC1 and E2F-signalling pathways were downregulated following CBX2 knockdown and that in MCF-7 and T47D cells, RBL2 expression was elevated following CBX2 depletion. This suggests that the pro-oncogenic role of CBX2 may have commonalities across BCa subtypes. Investigation of patient gene expression data sets also identified a potential role for CBX2 inhibiting RBL2 expression and, therefore, DREAM complex activity in other cancer types, again suggesting a common CBX2-associated oncogenic function.

## 5. Conclusions

In summary, this study has shown that CBX2 plays a crucial role in TNBC cell growth and viability. For the first time in BCa, we have directly identified a putative CBX2-regulated gene signature that has highlighted oncogenic signalling pathways reliant on CBX2 expression in both TNBC and ER+ve subtypes. In addition, we have provided putative mechanisms through which CBX2 promotes these oncogenic pathways, most notably, the inhibition of DREAM complex activity via repression of the tumour suppressor gene *RBL2*. Identification of the mechanisms by which CBX2 promotes BCa growth will provide biomarkers of CBX2 activity and further our understanding of cPRC1 as an oncogenic complex. Aggressive disease such as TNBC, which has few therapeutic options, desperately requires novel therapeutic targets, for which CBX2 is a promising candidate. Although ER+ve disease has targeted therapeutic options, many cancers become resistant to first-line therapy [1]; therefore, additional target proteins, such as CBX2, could still be efficacious in treating this BCa subtype. Importantly we have also demonstrated the requirement for CBX2–chromatin interactions for cell growth and provided data for the first time, showing the utility of a selective CBX2 inhibitor to slow TNBC cell growth. Future use of CBX2 inhibitors and definition of the global CBX2-binding profile in BCa will advance our understanding of the oncogenic role of CBX2 and further validate CBX2 as a genuine therapeutic target.

## Figures and Tables

**Table 1:** Significantly enriched gene sets for genes downregulated following CBX2 depletion using siCBX2#3 in MDA-MB-231 cells. The 10 most significantly enriched gene sets from the Hallmark, Gene Ontology, and Oncogenic signature curated gene set database are shown. Gene sets are inversely ranked by normalised enrichment score (NES). SIZE = number of differentially expressed genes enriched in the gene set. NOM p-val = nominal *p*-value, FDR q-val = false discover rate q-value.

Hallmark Gene Set				
**NAME**	**SIZE**	**NES**	**NOM *p*-val**	**FDR q-val**
HALLMARK_G2M_CHECKPOINT	49	−4.395	<0.001	<0.001
HALLMARK_E2F_TARGETS	45	−4.221	<0.001	<0.001
HALLMARK_XENOBIOTIC_METABOLISM	48	−2.373	<0.001	0.005
HALLMARK_ESTROGEN_RESPONSE_LATE	48	−2.173	0.004	0.018
HALLMARK_APOPTOSIS	46	−2.106	0.006	0.023
HALLMARK_MITOTIC_SPINDLE	46	−1.989	0.006	0.037
HALLMARK_MTORC1_SIGNALING	45	−1.902	0.012	0.050
HALLMARK_ESTROGEN_RESPONSE_EARLY	56	−1.866	0.010	0.054
HALLMARK_UV_RESPONSE_UP	33	−1.864	0.008	0.048
HALLMARK_MYC_TARGETS_V1	19	−1.853	0.010	0.046
Gene Ontology Gene Set				
**NAME**	**SIZE**	**NES**	**NOM *p*-val**	**FDR q-val**
GO_NEUROGENESIS	288	−3.522	<0.001	<0.001
GO_CHROMOSOME_ORGANIZATION	184	−3.492	<0.001	<0.001
GO_POSITIVE_REGULATION_OF_RESPONSE_TO_STIMULUS	390	−3.478	<0.001	<0.001
GO_CELL_CYCLE	233	−3.416	<0.001	<0.001
GO_REGULATION_OF_CELL_PROLIFERATION	336	−3.369	<0.001	<0.001
GO_POSITIVE_REGULATION_OF_GENE_EXPRESSION	347	−3.362	<0.001	<0.001
GO_CELL_PROLIFERATION	134	−3.360	<0.001	<0.001
GO_NEGATIVE_REGULATION_OF_DEVELOPMENTAL_PROCESS	168	−3.295	<0.001	<0.001
GO_POSITIVE_REGULATION_OF_CELL_PROLIFERATION	185	−3.286	<0.001	<0.001
GO_CELL_DIVISION	96	−3.240	<0.001	<0.001
Oncogenic Signature Gene Set				
**NAME**	**SIZE**	**NES**	**NOM *p*-val**	**FDR q-val**
RPS14_DN.V1_DN	32	−3.631	<0.001	<0.001
E2F3_UP.V1_UP	45	−3.291	<0.001	<0.001
PRC2_EZH2_UP.V1_DN	46	−2.782	<0.001	0.002
LEF1_UP.V1_UP	47	−2.593	<0.001	0.005
CSR_LATE_UP.V1_UP	47	−2.565	<0.001	0.005
NFE2L2.V2	87	−2.331	<0.001	0.015
HOXA9_DN.V1_DN	43	−2.244	<0.001	0.024
BMI1_DN.V1_DN	37	−2.179	0.004	0.031
CORDENONSI_YAP_CONSERVED_SIGNATURE	20	−2.140	0.002	0.035
KRAS.600_UP.V1_DN	53	−2.099	0.004	0.040

## Data Availability

The RNA sequencing data generated during the current study are available in the NCBI’s Gene Expression Omnibus repository (GEO-https://www.ncbi.nlm.nih.gov/geo/; accession number GSE198420 accessed 11 March 2022). All other data presented in this study are available in the published article and supplementary information.

## Supplementary Materials

The following supporting information can be downloaded at: https://www.mdpi.com/article/10.3390/cancers14143491/s1, Table S1: siRNA sequences. Table S2: Antibody details and uses. Table S3: Gene expression and ChIP/CUT&RUN qPCR primers. Table S4: Significantly enriched gene sets for genes downregulated following CBX2 depletion using siCBX2#2 in MDA-MB-231 cells. Table S5: Significantly enriched gene sets for genes downregulated following CBX2 depletion using siCBX2#3 in MCF-7 cells. Figure S1: Percentage of cells in the G2/M phase of the cell cycle following propidium iodide flow cytometry analysis of MDA-MB-231 and MDA-MB-468 cells. Figure S2: Ectopic CBX2 protein expression. Figure S3: MDA-MB-468 cell growth rescue experiment. Figure S4: CBX2 protein expression following SW2_152F treatment. Figure S5: GSEA analysis of CBX2-depleted MDA-MB-231 cells–MYC Targets. Figure S6: GSEA analysis of CBX2-depleted MDA-MB-231 cells–RBL2 targets. Figure S7: GSEA of genes significantly positively correlated with CBX2 expression from TCGA PanCancer Database for Breast Cancer. Figure S8: Propidium iodide flow cytometry analysis of MCF-7 and T47D cells. Figure S9: TSC1 expression in CBX2 depleted MCF-7 cells. Uncropped blots and densitometry intensity ratios.
