# Supplementary material for: The Epigenetic Regulatory Protein CBX2 Promotes mTORC1 Signalling and Inhibits DREAM Complex Activity to Drive Breast Cancer Cell Growth
Source: Cancers (Basel). 2022 Jul 18;14(14):3491. doi: 10.3390/cancers14143491 (PMC9321755; doi:10.3390/cancers14143491)
Supplement: Supplementary file 1 [file cancers-14-03491-s001.zip › cancers-1799272-supplementary.pdf]

Supplementary Table S1 – siRNA sequences

siRNA Name	Sequence (5' - 3')
siSCR	UUCUCCGAACGUGUCACGU
siCBX2#1	AGGAGGUGCAGAACCGGAA
siCBX2#2	GCAAGGGCAAGCUGGAGUA
siCBX2#3	CAAGGAAGCUCACUGCCAU

Supplementary Table S2 – Antibody details and uses

	Use	
**Antibody**	**Western Analysis**	**ChIP/CUT&RUN**
CBX2 - ab80044 - Abcam	X	
CBX2 - C15410339 - Diagenode		X
α-tubulin - 66031-1-Ig - Proteintech	X	
RBL2 - 13610S - CST	X	X
Rabbit IgG - C15410206 - Diagenode		X
**Secondary Antibodies**		
Polyclonal Swine Anti-Rabbit HRP - DAKO	X	
Polyclonal Rabbit Anti-Mouse HRP - DAKO	X	

Supplementary Table S3 – Gene expression and ChIP/CUT&RUN qPCR primers

**mRNA Primers**	**Sequence 5'-3'**
RPL13A F	CCTGGAGGAGAAGAGGAAAGAGA
RPL13A R	TTGAGGACCTCTGTGTATTTGTCAA
CBX2 F	GCTCCAAAGCCAGACTAACA
CBX2 R	CAGGGACAGACATCCTCATTTC
AURKA F	CCTACAAAAGAATATCACGGG
AURKA R	CAAGTACTTCTCTGAGCATTG
PLK1 F	ATTTCCGCAATTACATGAGC
PLK1 R	TCCTGGAAGAAGTTGATCTG
CCNA2 F	AGCTGCCTTTCATTTAGCACTCTAC
CCNA2 R	TTAAGACTTTCCAGGGTATATCCAGTC

**ChIP/CUT&RUN Primers**	**Sequence 5'-3'**
TSC1 F	GCCGTCTATCCTTCCTTTCGA
TSC1 2	CGCCAGGAAAAAGAGTCCC
PRKAA2 F	TTCCCTTTTACAGCCCCTCG
PRKAA2 R	TGGAAGAAGAGACGGGCCT
RBL2 F	CATGATTTTTGGCCCCCTTGA
RBL2 R	CAGGCACCCGTAGTCTTGA
CCNA2 F	AGTTCAAGTATCCCGCGACT
CCNA2 R	GGTTTACCCTTCACTCGCCT
UBE2C F	GGACCGTTTGAATGAGACGC
UBE2C R	CCCAGGAAGACCGTTAGTCG
CCNB1 F	CTGGAAACGCATTCTCTGCG
CCNB1 R	GCCAGCCTAGCCTCAGATTT

Supplementary Table S4 – Significantly enriched gene sets for genes downregulated following CBX2 depletion using siCBX2#2 in MDA-MB-231 cells. Enriched gene sets from the Hallmark and Oncogenic Signature curated datasets are shown. Gene sets are inversely ranked by Normalised Enrichment Score (NES). SIZE = number of differentially expressed genes enriched in the gene set. NOM = nominal p-value, FDR = False Discover Rate q-value.

Hallmark Gene Set				
**NAME**	**SIZE**	**NES**	**NOM p-val**	**FDR q-val**
HALLMARK_INTERFERON_GAMMA_RESPONSE	46	-4.067	<0.001	<0.001
HALLMARK_TNFA_SIGNALING_VIA_NFKB	60	-3.603	<0.001	<0.001
HALLMARK_INFLAMMATORY_RESPONSE	43	-3.260	<0.001	<0.001
HALLMARK_INTERFERON_ALPHA_RESPONSE	29	-2.982	<0.001	<0.001
HALLMARK_IL6_JAK_STAT3_SIGNALING	25	-2.853	<0.001	<0.001
HALLMARK_G2M_CHECKPOINT	40	-2.618	<0.001	<0.001
HALLMARK_MTORC1_SIGNALING	46	-2.435	<0.001	0.001
HALLMARK_KRAS_SIGNALING_UP	48	-2.392	0.002	0.002
HALLMARK_ALLOGRAFT_REJECTION	39	-2.339	0.004	0.002
HALLMARK_UV_RESPONSE_UP	40	-2.259	0.000	0.003
HALLMARK_MYC_TARGETS_V1	33	-2.223	0.002	0.004
HALLMARK_ANDROGEN_RESPONSE	26	-2.085	0.002	0.009
HALLMARK_ESTROGEN_RESPONSE_LATE	55	-1.953	0.010	0.018
HALLMARK_CHOLESTEROL_HOMEOSTASIS	20	-1.898	0.010	0.023
HALLMARK_XENOBIOTIC_METABOLISM	46	-1.896	0.004	0.022
HALLMARK_E2F_TARGETS	37	-1.767	0.014	0.041
Oncogenic signature gene set				
**NAME**	**SIZE**	**NES**	**NOM p-val**	**FDR q-val**
KRAS.BREAST_UP.V1_UP	18	-3.478	<0.001	<0.001
RPS14_DN.V1_UP	56	-3.447	<0.001	<0.001
KRAS.600.LUNG.BREAST_UP.V1_UP	53	-3.299	<0.001	<0.001
KRAS.LUNG.BREAST_UP.V1_UP	26	-3.152	<0.001	<0.001
BMI1_DN.V1_UP	42	-2.479	<0.001	0.005
MEL18_DN.V1_DN	33	-2.311	0.002	0.014
LEF1_UP.V1_DN	52	-2.301	<0.001	0.014
MTOR_UP.V1_UP	40	-2.282	<0.001	0.014
PRC2_EED_UP.V1_DN	46	-2.194	<0.001	0.022
JNK_DN.V1_UP	42	-2.186	0.002	0.021

Supplementary Table S5 – Significantly enriched gene sets for genes downregulated following CBX2 depletion using siCBX2#3 in MCF-7 cells. The 10 most significantly enriched gene sets from the Hallmark and Oncogenic Signature curated datasets shown. Gene sets inversely ranked by Normalised Enrichment Score (NES). NOM = nominal p-value, FDR = False Discover Rate q-value.

Hallmark Gene Set				
**NAME**	**SIZE**	**NES**	**NOM p-val**	**FDR q-val**
HALLMARK_E2F_TARGETS	121	-7.096	<0.001	<0.001
HALLMARK_G2M_CHECKPOINT	112	-6.241	<0.001	<0.001
HALLMARK_MYC_TARGETS_V1	75	-5.580	<0.001	<0.001
HALLMARK_ESTROGEN_RESPONSE_LATE	94	-4.446	<0.001	<0.001
HALLMARK_ESTROGEN_RESPONSE_EARLY	90	-4.059	<0.001	<0.001
HALLMARK_MYC_TARGETS_V2	33	-3.763	<0.001	<0.001
HALLMARK_MTORC1_SIGNALING	61	-3.199	<0.001	<0.001
HALLMARK_KRAS_SIGNALING_UP	56	-2.839	<0.001	<0.001
HALLMARK_MITOTIC_SPINDLE	92	-2.477	<0.001	0.002
HALLMARK_OXIDATIVE_PHOSPHORYLATION	55	-2.451	<0.001	0.002
Oncogenic signature gene set				
**NAME**	**SIZE**	**NES**	**NOM p-val**	**FDR q-val**
CSR_LATE_UP.V1_UP	75	-4.743	<0.001	<0.001
RPS14_DN.V1_DN	84	-4.577	<0.001	<0.001
MYC_UP.V1_UP	70	-3.761	<0.001	<0.001
VEGF_A_UP.V1_DN	85	-3.551	<0.001	<0.001
RB_P107_DN.V1_UP	65	-3.484	<0.001	<0.001
PRC2_EZH2_UP.V1_DN	76	-3.468	<0.001	<0.001
HOXA9_DN.V1_DN	64	-3.289	<0.001	<0.001
MEK_UP.V1_UP	81	-3.282	<0.001	<0.001
GCNP_SHH_UP_LATE.V1_UP	64	-3.170	<0.001	<0.001
E2F1_UP.V1_UP	78	-3.161	<0.001	<0.001

A

MDA-MB-231

	siCR		siCBX2#1		siCBX2#2		siCBX2#3	
Cell Cycle Phase	Mean % of cells	SD	Mean % of cells	SD	Mean % of cells	SD	Mean % of cells	SD
Sub-G1	10.7	2.8	10.8	0.9	17.4	0.7	22.4	4.3
G1	58.3	1.0	51.9	0.9	31.6	1.6	38.1	2.1
S	19.8	0.8	19.2	0.4	37.7	1.4	13.4	0.4
G2M	11.7	1.1	18.5	0.3	13.9	1.0	26.5	1.9

MDA-MB-468

	siCR		siCBX2#1		siCBX2#2		siCBX2#3	
Cell Cycle Phase	Mean % of cells	SD	Mean % of cells	SD	Mean % of cells	SD	Mean % of cells	SD
Sub-G1	5.7	2.8	5.5	2.7	8.7	2.9	16.5	6.7
G1	60.0	3.2	63.9	0.5	58.9	5.7	41.2	21.2
S	14.2	1.9	12.4	1.5	13.3	2.2	13.8	1.8
G2M	18.8	2.6	17.0	3.6	19.4	9.2	26.3	14.9

B

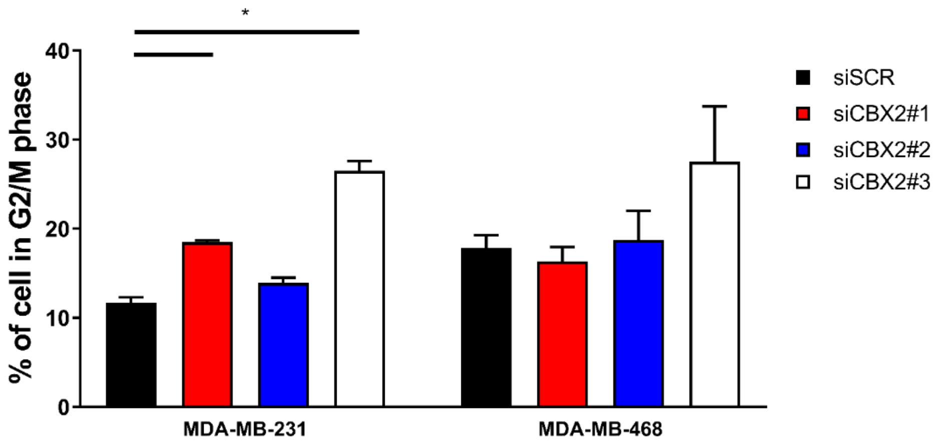

Supplementary Figure S1 – Percentage of cells in G2/M phase of the cell cycle following propidium iodide flow cytometry analysis of MDA-MB-231 and MDA-MB-468 cells. MDA-MB-231 and MDA-MB-468 cells were harvested and stained with propidium iodide 72 hours post transfection for flow cytometry analysis. (A) Table of flow cytometry data showing percentage of cells in each phase of the cell cycle. (B) Percentage of cells in G2/M phase of the cell cycle. Data are an average of 3 repeats ± SEM and comparisons of the percentage of cells in each cell cycle phase were made between each transfection condition. *P*-values were determined by Turkey's multiple comparisons test (*denotes $P < 0.05$).

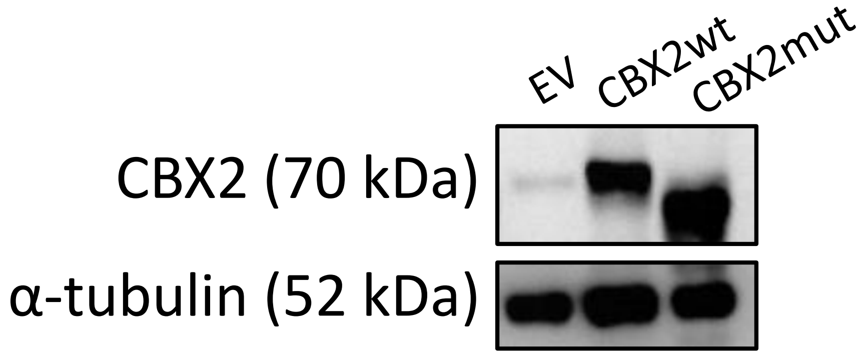

Supplementary Figure S2 – Ectopic CBX2 protein expression. MDA-MB-231 cells were transfected with 1 µg of either an empty vector control plasmid (EV), a plasmid containing a wild-type version of CBX2 (CBX2wt) or a chromodomain deficient mutant (CBX2mut) and grown for 72 hours followed by western blot analysis using antibodies specific to CBX2 and α-tubulin. α-tubulin was used to compare protein loading between samples. The band height for CBX2mut is lower than the wild type version of CBX2 due to an N-terminal deletion of the chromodomain of CBX2.

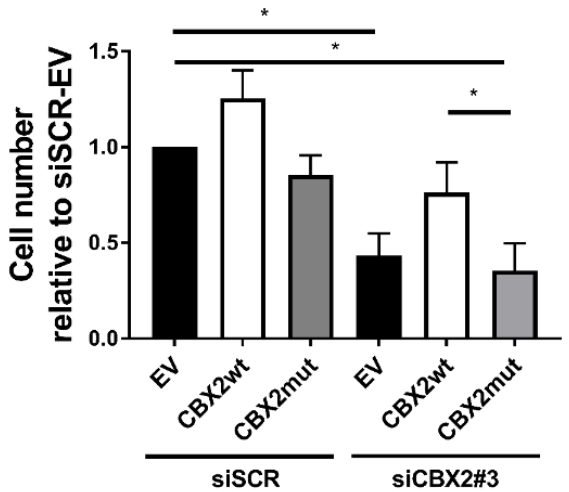

Supplementary Figure S3 – MDA-MB-468 cell growth rescue experiment. MDA-MB-468 cells were co-transfected with combinations of siSCR, siCBX2#3 and empty vector (EV) or CBX2wt/mut plasmids and grown for 72 hours followed by cell counts. Data are the average of three independent experiments ± SEM and is expressed relative to cell counts for siSCR and empty vector co-transfected cells. *P*-values were determined by Turkey's multiple comparisons test (*denotes $P < 0.05$).

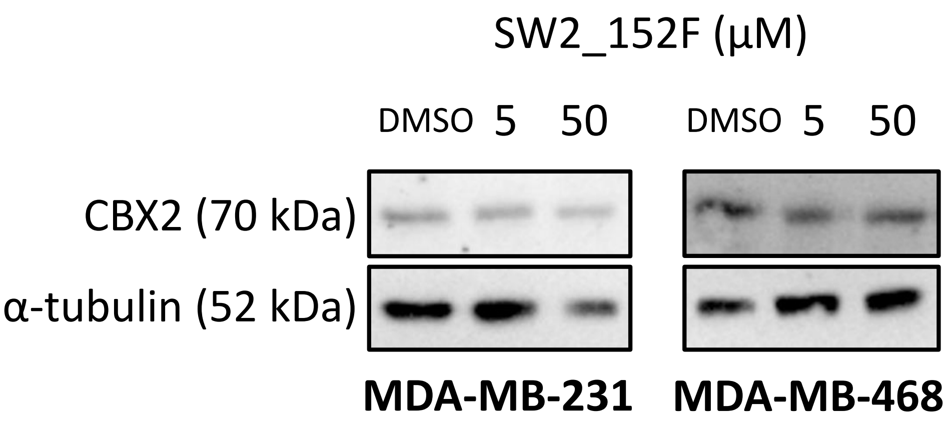

Supplementary Figure S4 – CBX2 protein expression following SW2_152F treatment. MDA-MB-231 and MDA-MB-468 cells were treated with a DMSO control or 5 and 50 µM of SW2_152F and grown for 72 hours (MDA-MB-231) or 96 hours (MDA-MB-468) followed by western blot analysis using antibodies specific to CBX2 and α-tubulin. α-tubulin was used to compare protein loading between samples.

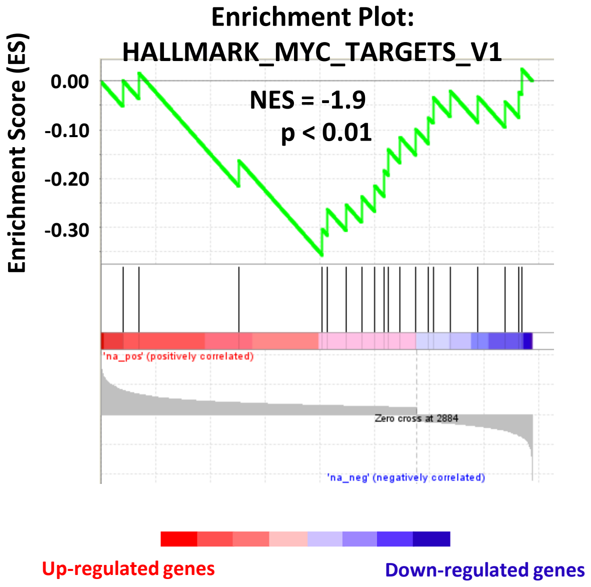

Supplementary Figure S5 - GSEA analysis of CBX2-depleted MDA-MB-231 cells – MYC Targets. MDA-MB-231 cells were transfected with either a non-silencing control siRNA (siSCR) or siCBX2#3 and grown for 72 hours. RNA was extracted and RNA-sequencing analysis was performed. GSEA of the CBX2-regulated transcriptome (defined as genes significantly (padj<0.05) up (n=2884) or down (n=1060) regulated 1.5 fold after knockdown compared to siSCR controls) against the "Hallmark" curated dataset identified significant negative enrichment of genes associated with the HALLMARK_MYC_TARGETS_V1 gene set. NES = Normalised Enrichment Score. p= nominal p-value.

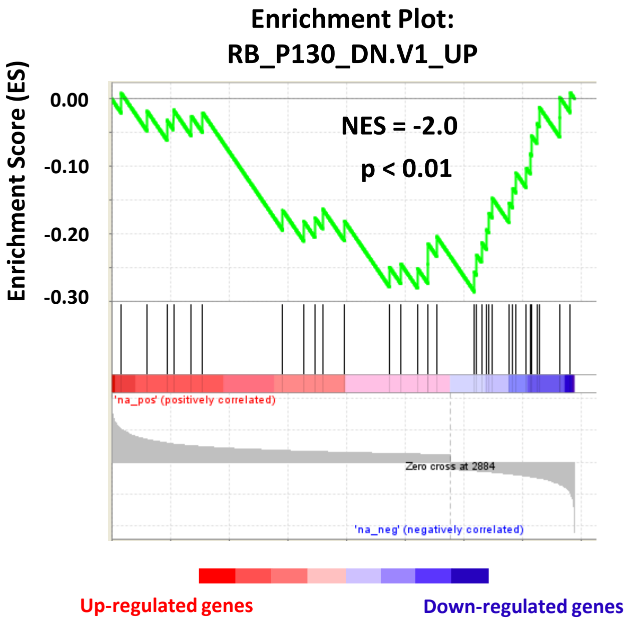

Supplementary Figure S6 - GSEA analysis of CBX2-depleted MDA-MB-231 cells – RBL2 targets. MDA-MB-231 cells were transfected with either a non-silencing control siRNA (siSCR) or siCBX2#3 and grown for 72 hours. RNA was extracted and RNA-sequencing analysis was performed. GSEA of the CBX2-regulated transcriptome (defined as genes significantly (padj<0.05) up (n=2884) or down (n=1060) regulated 1.5 fold after knockdown compared to siSCR controls) against the "Hallmark" curated dataset identified significant negative enrichment of genes associated with the RB_P130_DN.V1_UP gene set. This gene set represents genes upregulated when RBL2 expression is low. NES = Normalised Enrichment Score. p= nominal p-value.

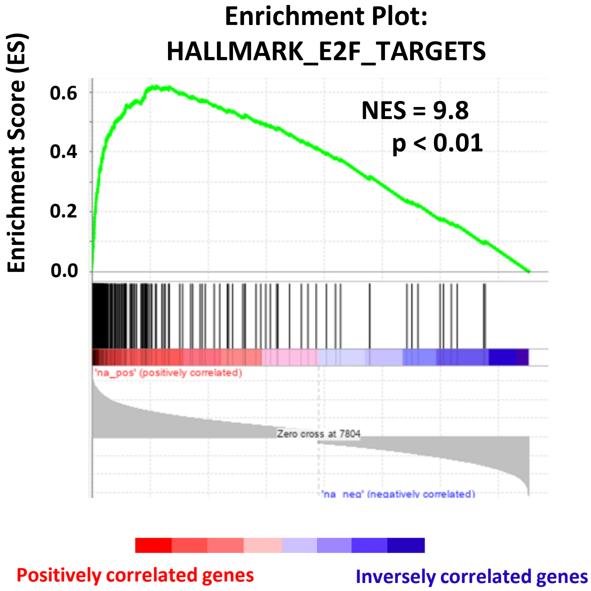

Supplementary Figure S7 - GSEA of genes significantly positively correlated (Spearmans p < 0.05) with CBX2 expression from TCGA PanCancer Database for Breast Cancer. NES = Normalised Enrichment Score. p= nominal p-value.

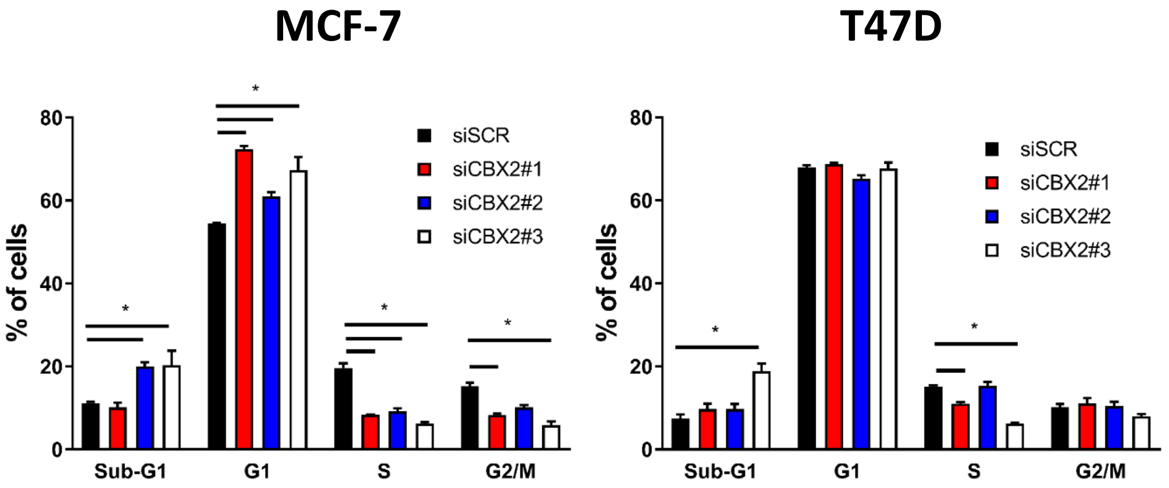

<u>MCF-7</u>

	siSCR		siCBX2#1		siCBX2#2		siCBX2#3	
Cell Cycle Phase	Mean % of cells	SD	Mean % of cells	SD	Mean % of cells	SD	Mean % of cells	SD
Sub-G1	11.2	0.5	10.1	2.0	20.0	1.7	20.3	6.0
G1	54.5	0.2	72.4	1.3	61.0	1.8	67.3	5.5
S	19.7	1.9	8.4	0.1	9.2	1.2	6.2	0.7
G2M	15.2	1.6	8.4	0.5	10.1	1.0	5.9	1.4

<u>T47D</u>

	siSCR		siCBX2#1		siCBX2#2		siCBX2#3	
Cell Cycle Phase	Mean % of cells	SD	Mean % of cells	SD	Mean % of cells	SD	Mean % of cells	SD
Sub-G1	7.4	1.8	9.8	2.3	9.8	2.1	18.9	3.1
G1	68.0	0.9	68.7	0.5	65.2	1.4	67.7	2.6
S	15.0	0.7	11.0	0.6	15.3	1.6	6.2	0.4
G2M	10.2	1.4	11.1	2.2	10.5	1.8	8.1	0.9

Supplementary Figure S8 – Propidium iodide flow cytometry analysis of MCF-7 and T47D cells. MCF-7 and T47D cells were harvested and stained with propidium iodide 72 hours post transfection for flow cytometry analysis. Data are an average of 3 repeats ± SEM and comparisons of the percentage of cells in each cell cycle phase were made between each transfection condition. *P*-values were determined by Turkey's multiple comparisons test (*denotes $P < 0.05$).

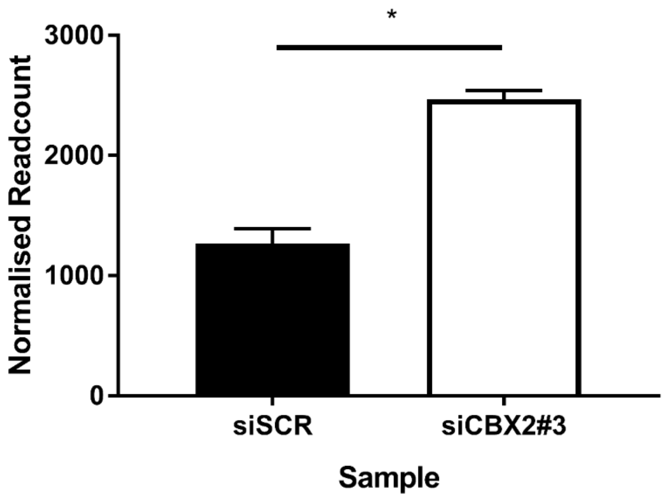

Supplementary Figure S9 – TSC1 expression in CBX2 depleted MCF-7 cells. MCF-7 cells were transfected with either a non-silencing control siRNA (siSCR) or siCBX2#3 and grown for 72 hours. RNA was extracted and RNA-sequencing analysis was performed. *TSC1* gene expression shown as mean normalised read count. Data is an average of 3 independent experiments ± SEM (* padj < 0.05).

Western Blot Images
Uncropped blots and densitometry intensity ratios

Figure 1a – Red box indicates part of image in figure. Intensity ratios relative to siSCR transfected cells (normalised to 100%) are shown below each band. Sample is indicated above each band. IB = immunoblot of designated protein.

MDA-MB-231

IB: CBX2

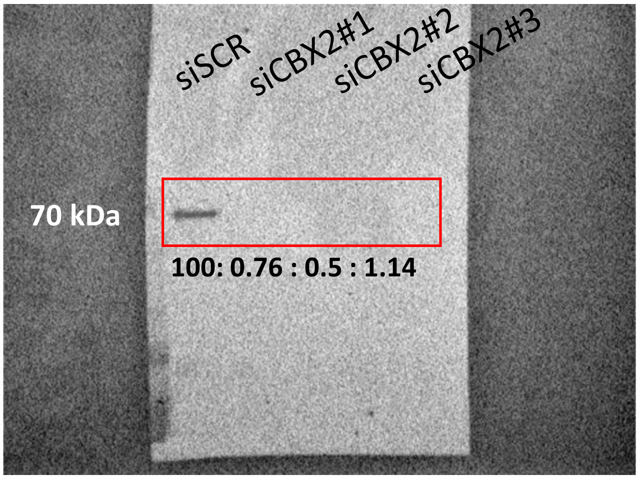

IB: α-tubulin

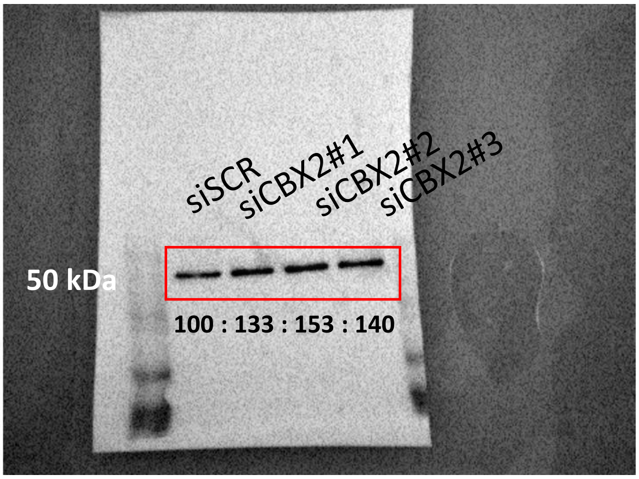

MDA-MB-468

IB: CBX2

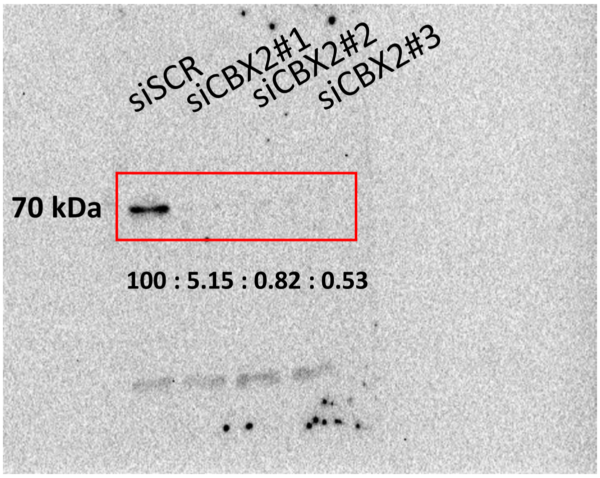

IB: α-tubulin

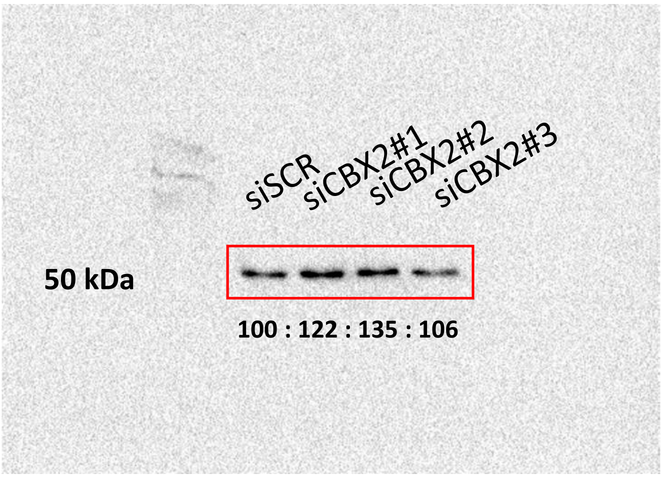

Hs-578T

IB: CBX2

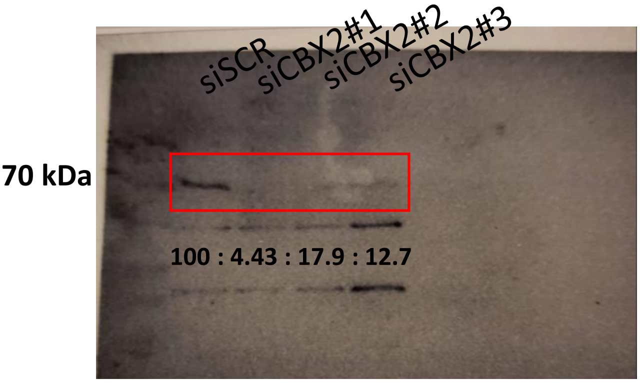

IB: α-tubulin

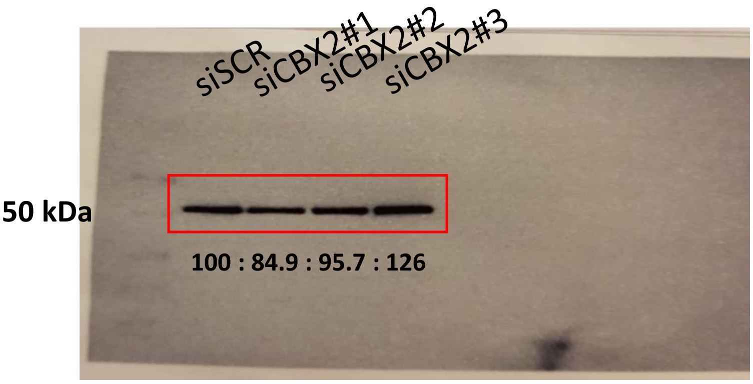

Figure 2c - Red box indicates part of image in figure. Intensity ratios relative to siSCR transfected cells (normalised to 100%) are shown below the red box or blot. Sample is indicated above each band. IB = immunoblot of designated protein.

MDA-MB-231

IB: RBL2

130 kDa

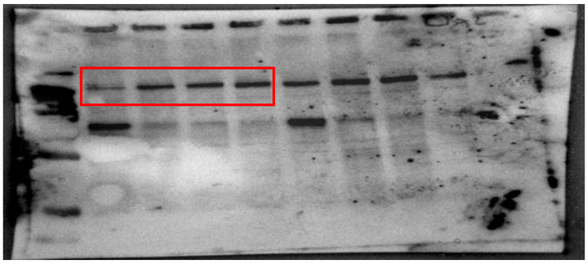

100 : 172 : 183 : 191

IB: CBX2

70 kDa

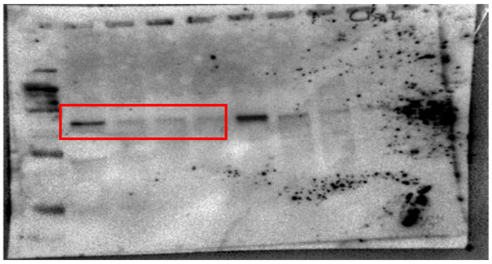

100 : 20.6 : 19.9 : 16.6

IB: α-tubulin

50 kDa

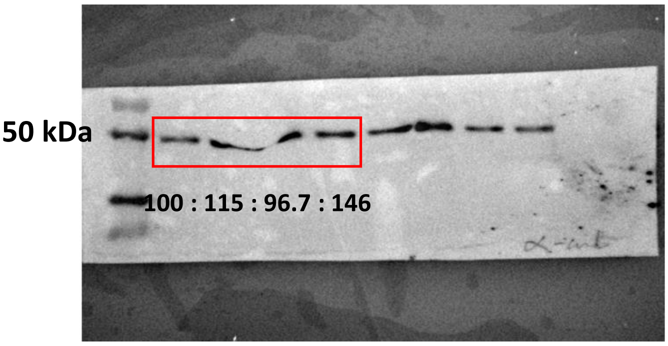

Lane order left to right of samples shown in figure (red box):

1. siSCR
2. siCBX2#1
3. siCBX2#2
4. siCBX2#3

MDA-MB-468

IB: RBL2

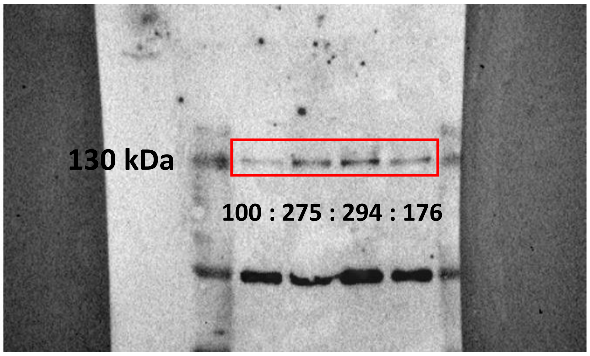

IB: CBX2

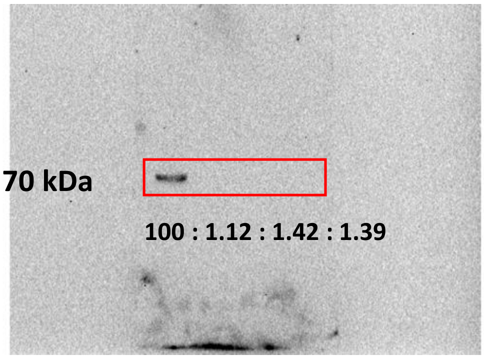

IB: α-tubulin

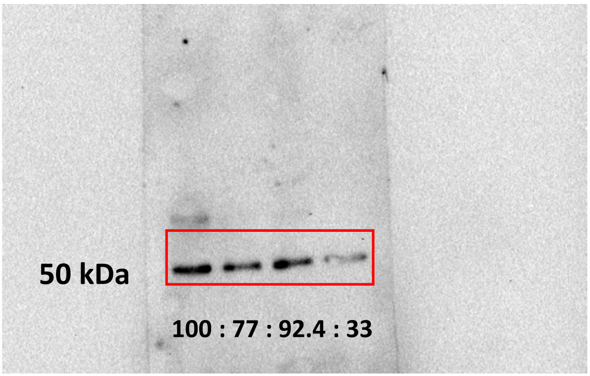

Lane order left to right of samples shown in figure (red box):

1. siSCR
2. siCBX2#1
3. siCBX2#2
4. siCBX2#3

Figure 4a - Red box indicates part of image in figure. Intensity ratios relative to siSCR transfected cells (normalised to 100%) are shown below the red box or blot. Sample is indicated above each band. IB = immunoblot of designated protein.

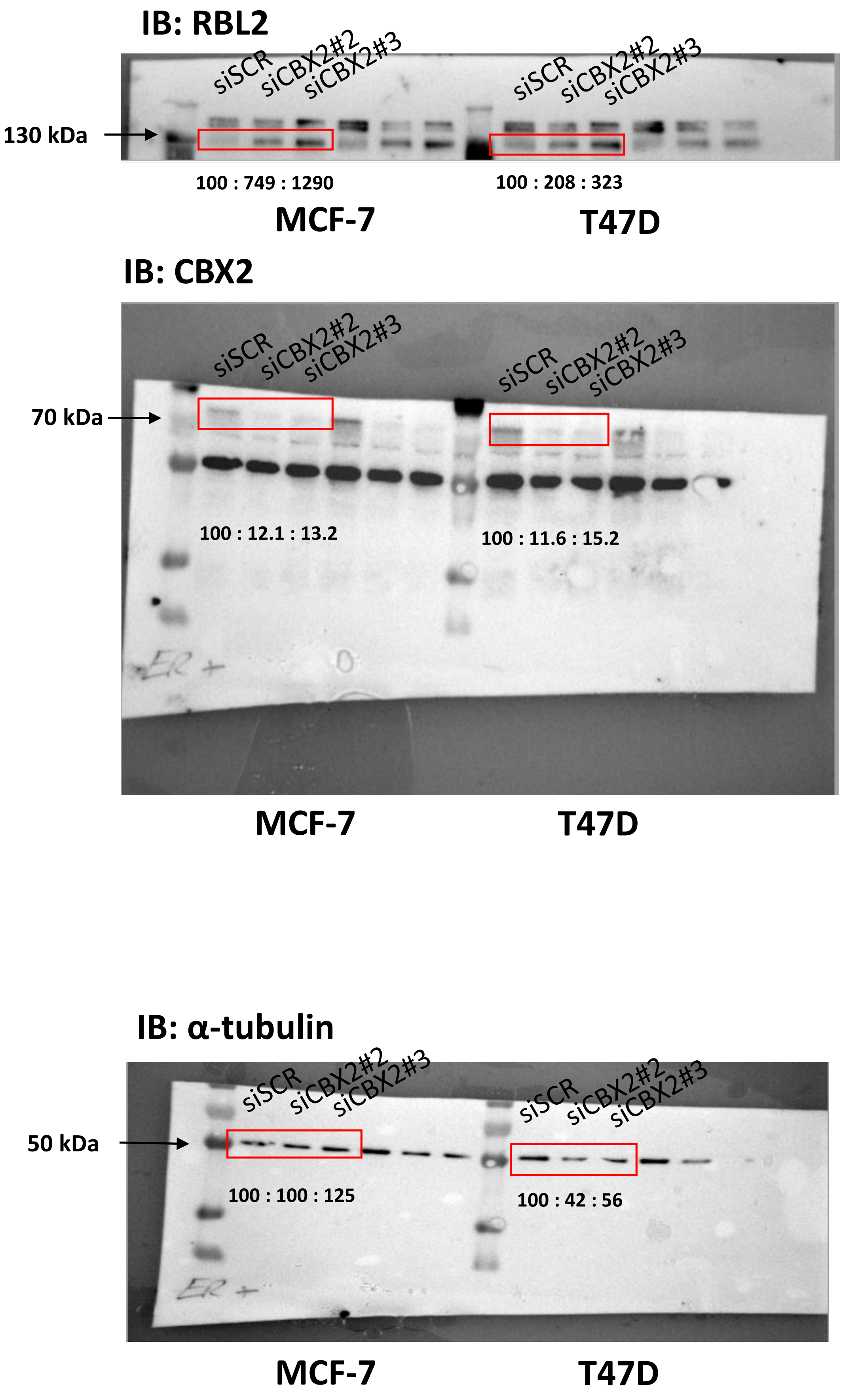

Supplementary Figure S2 - Red box indicates part of image in figure. Intensity ratios relative to empty vector (EV) transfected cells (normalised to 100%) are shown below the red box or blot. Sample is indicated above each band. IB = immunoblot of designated protein.

IB: CBX2

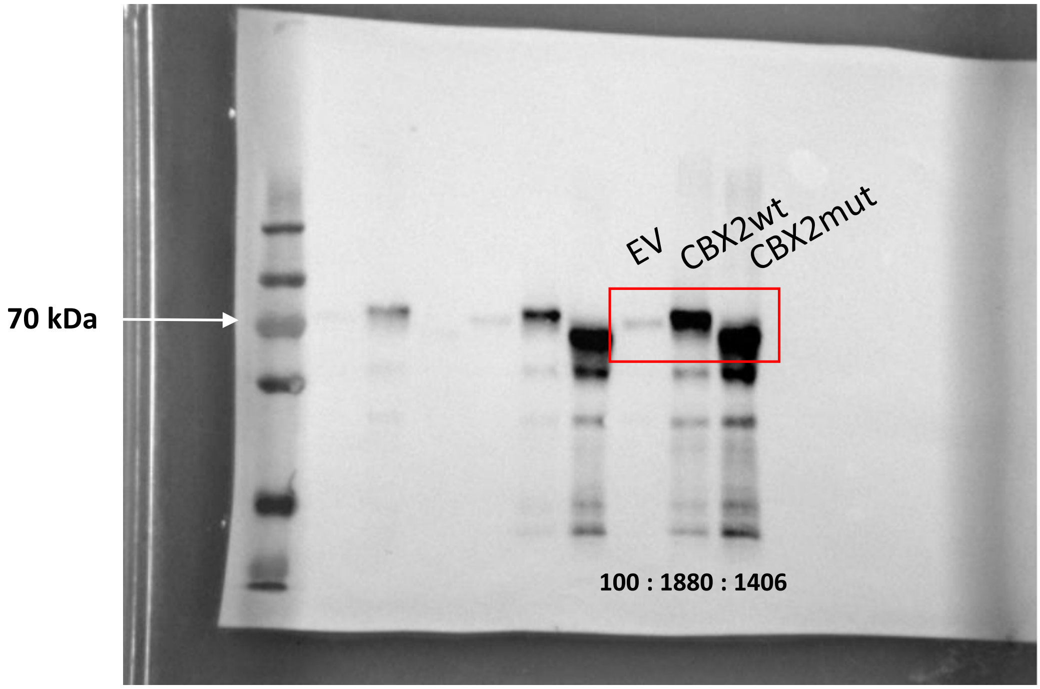

IB: α-tubulin

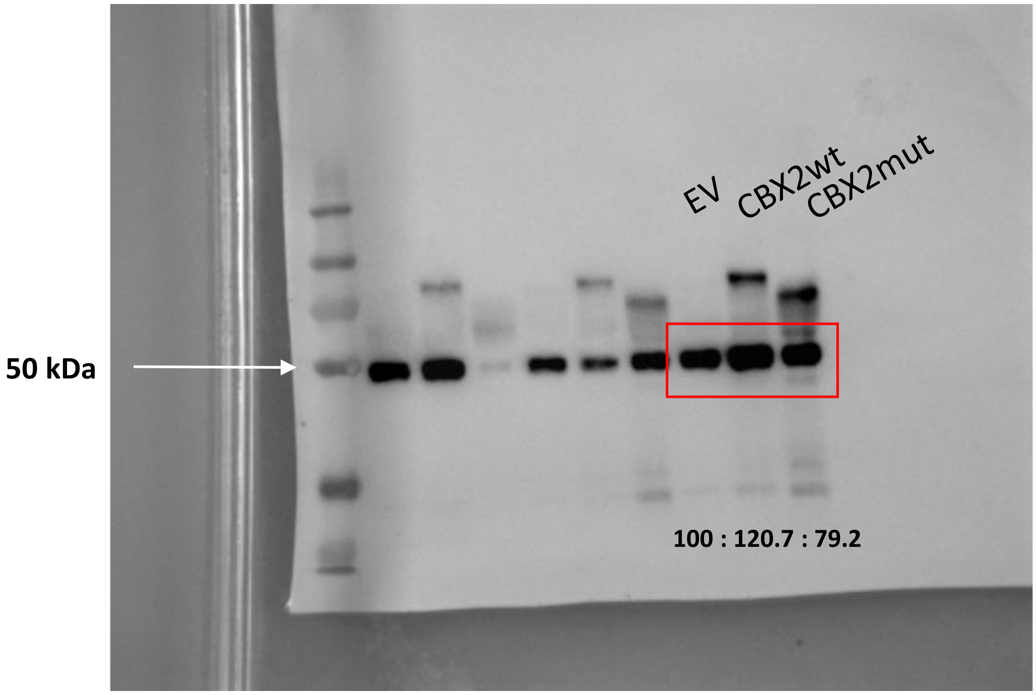

Supplementary Figure S4 - Red box indicates part of image in figure. Intensity ratios relative to DMSO treated cells (normalised to 100%) are shown below the red box or blot. Sample is indicated above each band. IB = immunoblot of designated protein.

MDA-MB-231

IB: CBX2

70 kDa

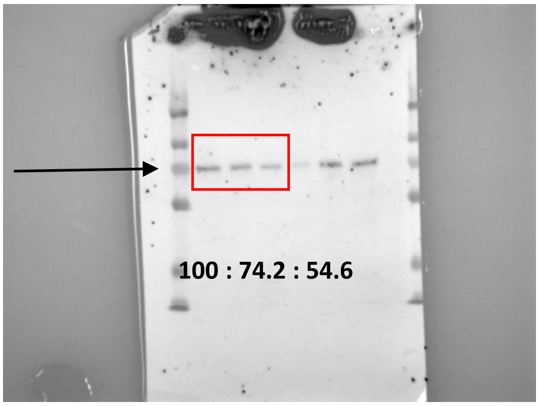

IB: α-tubulin

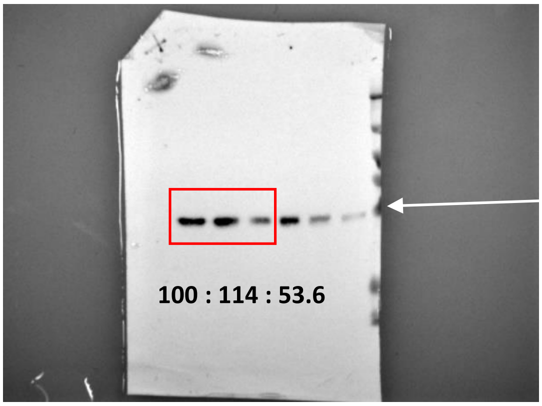

50 kDa

Lane order left to right of samples shown in figure (red box):

1. DMSO
2. 5 µM SW2_152F
3. 50 µM SW2_152F

MDA-MB-468

IB: CBX2

70 kDa

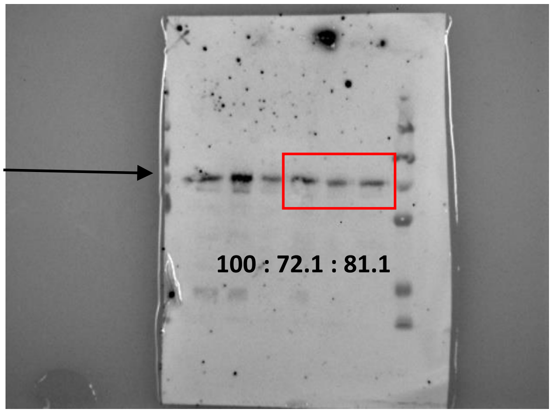

IB: α-tubulin

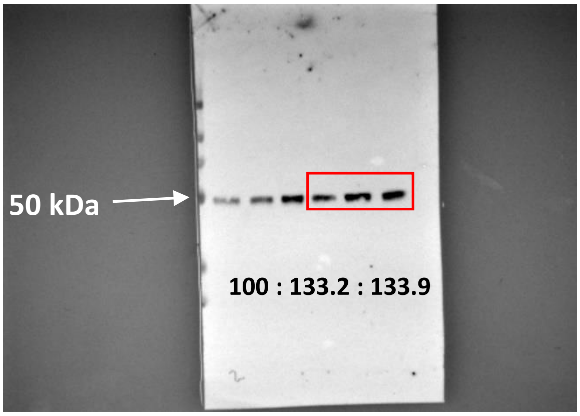

Lane order left to right of samples shown in figure (red box):

1. DMSO
2. 5 µM SW2_152F
3. 50 µM SW2_152F
